# PTEN regulates glioblastoma oncogenesis through chromatin-associated complexes of DAXX and histone H3.3

**DOI:** 10.1038/ncomms15223

**Published:** 2017-05-12

**Authors:** Jorge A. Benitez, Jianhui Ma, Matteo D’Antonio, Antonia Boyer, Maria Fernanda Camargo, Ciro Zanca, Stephen Kelly, Alireza Khodadadi-Jamayran, Nathan M. Jameson, Michael Andersen, Hrvoje Miletic, Shahram Saberi, Kelly A. Frazer, Webster K. Cavenee, Frank B. Furnari

**Affiliations:** 1 Ludwig Institute for Cancer Research, La Jolla, California 92093-0660, USA; 2 The Moores Cancer Center, University of California San Diego, La Jolla, California 92093, USA; 3 Department of Pediatrics and Rady Children’s Hospital, University of California San Diego, La Jolla, California 92093, USA; 4 Sanford Consortium for Regenerative Medicine, University of California, San Diego, 3855 Health Science Drive, La Jolla, California 92037, USA; 5 Department of Pathology, Laura & Isaac Perlmutter Cancer Center, and The Helen L. and Martin S. Kimmel Center for Stem Cell Biology, NYU School of Medicine, New York, New York 10016, USA; 6 Center for Health Informatics and Bioinformatics, NYU School of Medicine, New York, New York 10016, USA; 7 Department of Pathology, Haukeland University Hospital, 5021 Bergen, Norway; 8 Department of Biomedicine, University of Bergen, 5009 Bergen, Norway; 9 KG Jebsen Brain Tumour Research Center, University of Bergen, 5009 Bergen, Norway; 10 Department of Neurosciences, University of California, San Diego, La Jolla, California 92093, USA; 11 Institute for Genomic Medicine, University of California San Diego, La Jolla, California 92093, USA; 12 Department of Pathology, University of California San Diego, La Jolla, California 92093, USA

## Abstract

Glioblastoma (GBM) is the most lethal type of human brain cancer, where deletions and mutations in the tumour suppressor gene *PTEN* (phosphatase and tensin homolog) are frequent events and are associated with therapeutic resistance. Herein, we report a novel chromatin-associated function of PTEN in complex with the histone chaperone DAXX and the histone variant H3.3. We show that PTEN interacts with DAXX and, in turn PTEN directly regulates oncogene expression by modulating DAXX-H3.3 association on the chromatin, independently of PTEN enzymatic activity. Furthermore, *DAXX* inhibition specifically suppresses tumour growth and improves the survival of orthotopically engrafted mice implanted with human PTEN-deficient glioma samples, associated with global H3.3 genomic distribution changes leading to upregulation of tumour suppressor genes and downregulation of oncogenes. Moreover, *DAXX* expression anti-correlates with *PTEN* expression in GBM patient samples. Since loss of chromosome 10 and *PTEN* are common events in cancer, this synthetic growth defect mediated by *DAXX* suppression represents a therapeutic opportunity to inhibit tumorigenesis specifically in the context of PTEN deletion.

## Abstract (web summary)

*PTEN* mutations are frequent in glioblastoma and often are associated with therapeutic resistance. Here, the authors demonstrate that PTEN regulates gene expression at the chromatin level by interacting with the histone chaperone DAXX and H3.3, and that *DAXX* inhibition inhibits *PTEN*-deficient GBM growth *in vivo*.

Glioblastoma (GBM) is the most common and aggressive form of cancer of the central nervous system. The TCGA (The Cancer Genome Atlas) data indicate that about 50% of GBMs harbour somatic alterations in the phosphatidylinositol 3-OH kinase pathway^1^,^2^. One of the essential regulators of this pathway that is significantly altered in GBMs (30–40%) is the *PTEN* tumour suppressor gene^1^,^3^, which encodes a phosphatase responsible for the removal of phosphate from the 3′ position of the phospholipid second messenger phosphatidylinositol-3,4,5-trisphosphate, thus opposing mitogenic signalling mediated by class 1 phosphatidylinositol 3-OH kinases^4^. The loss of PTEN function has been mechanistically linked to metastasis^5^, and lack of radio-therapy^6^ and chemo-therapy^7^,^8^ response in brain and breast cancer patients, indicating that PTEN is a key regulator of tumour sensitivity to multiple therapeutic approaches. It has been well established that different epigenetic, transcriptional and post-translational mechanisms control the level and function of PTEN. Moreover, PTEN protein–protein interactions can also affect its tumour suppressor properties^9^,^10^,^11^.

GBMs undergo genetic lesions that affect the epigenomic machinery that controls histone modifications, DNA methylation and gene expression. One such target is the histone H3 variant, H3.3, which is incorporated into chromatin in a cell cycle independent manner and is associated with transcriptionally active and silent chromatin in somatic and embryonic cells^12^,^13^. Two distinct and mutually exclusive H3.3 mutations (K27M and G34R/V) have been identified in paediatric GBMs^14^,^15^, associated with global downregulation of the repressive histone mark H3K27me3, DNA hypomethylation^16^,^17^,^18^, ALT (Alternative Lengthening of Telomere) phenotype^14^ and upregulation of the MYCN pathway^19^. In adult GBMs, H3.3 expression is repressed by MLL5 (Mixed Lineage Leukemia 5), leading to chromatin reorganization and self-renewal^20^. Histone chaperones that are involved in the recruitment of H3.3 to chromatin are DAXX (death-domain associated protein), ATRX (alpha-thalassaemia/mental retardation X-linked syndrome protein) and HIRA (histone cell cycle regulator)^12^,^21^,^22^,^23^,^24^. Somatic mutations of *DAXX* and *ATRX* have been reported in adult pancreatic neuroendocrine tumours^25^, low grade gliomas^26^,^27^ and paediatric high grade gliomas^14^; however, genetic alterations of *DAXX* are rare in other types of cancers.

To date, various approaches have been used to target chromatin deregulation in cancer cells^28^,^29^,^30^. Here, we report a novel chromatin-associated function of the PTEN tumour suppressor that represses oncogene expression and tumour growth in patient-derived glioma xenografts through DAXX-H3.3 association. We show that DAXX physically interacts with PTEN, and PTEN regulates H3.3 loading on chromatin by limiting DAXX interactions with this histone, and thereby controls expression of several tumour-promoting genes. Moreover, *DAXX* inhibition affects global H3.3 deposition and gene expression, specifically suppresses intracranial tumour growth and significantly improves the survival of *PTEN-null* glioma-bearing mice. These results demonstrate a synthetic growth defect that occurs due to loss of these two tumour suppressor genes.

## Results

### PTEN interacts with DAXX and controls oncogene expression

**Figure 1:**
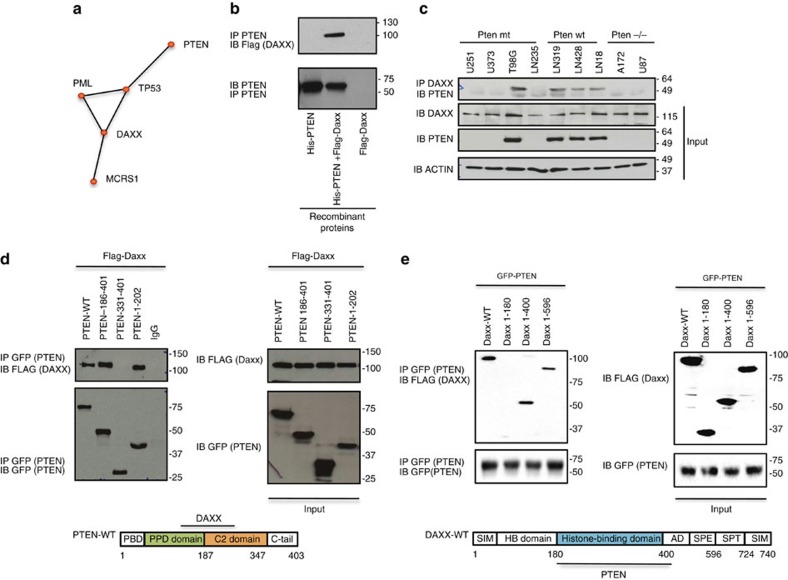
DAXX interacts with PTEN. (**a**) Predicted protein–protein interactions between PTEN, TP53, PML, DAXX and MCRS1 using the human protein interaction map. (**b**) *In vitro* pull-down using mixed Flag-DAXX and His-PTEN recombinant proteins immunoprecipitated (IP) with anti-PTEN and immunoblotted (IB) with anti-Flag. (**c**) Co-immunoprecipitation assay using whole cell protein lysates from different cell lines expressing endogenous DAXX and PTEN. IP anti-DAXX and IB anti-PTEN. (**d**,**e**) PTEN and DAXX interacting domains were mapped using 293 T cells co-transfected with Flag-Daxx-wt or GFP-PTEN and deletion constructs of each protein.

Several reports have shown that PTEN can control tumorigenesis independent of its enzymatic activity, through its interaction with specific nuclear proteins^9^,^10^,^11^. To uncover further PTEN-nuclear interactions, an *in silico* analysis was performed using the Human Interactome Map (HiMAP)^31^ bioinformatics site. From this analysis four interactome complexes were predicted for PTEN and for DAXX, that included previously reported interactions PTEN–TP53 (ref. 10), –MCRS1 (ref. 11) and –PML^9^ and DAXX–TP53 (ref. 32), –MCRS1 (ref. 33) and –PML^34^, and a new interaction consisting of PTEN–DAXX (Fig. 1a). Since DAXX is a key regulator of gene expression^35^,^36^ and has been shown to indirectly regulate PTEN stability^37^, we determined whether PTEN and DAXX could be physically associated. Pulldown assays using purified recombinant proteins (Fig. 1b) and total protein lysates from glioma cells that expressed different endogenous PTEN and DAXX levels (Fig. 1c) demonstrated that PTEN can physically interact with DAXX. To map the interaction domains for each protein, various GFP-PTEN and Flag-DAXX deletion constructs were co-transfected with full length Flag-DAXX or GFP-PTEN, respectively. These experiments showed that DAXX bound to the unfolded PTEN-hinge domain (amino acids 186–202) (Fig. 1d), while PTEN bound DAXX through its histone-binding domain (Fig. 1e).

**Figure 2:**
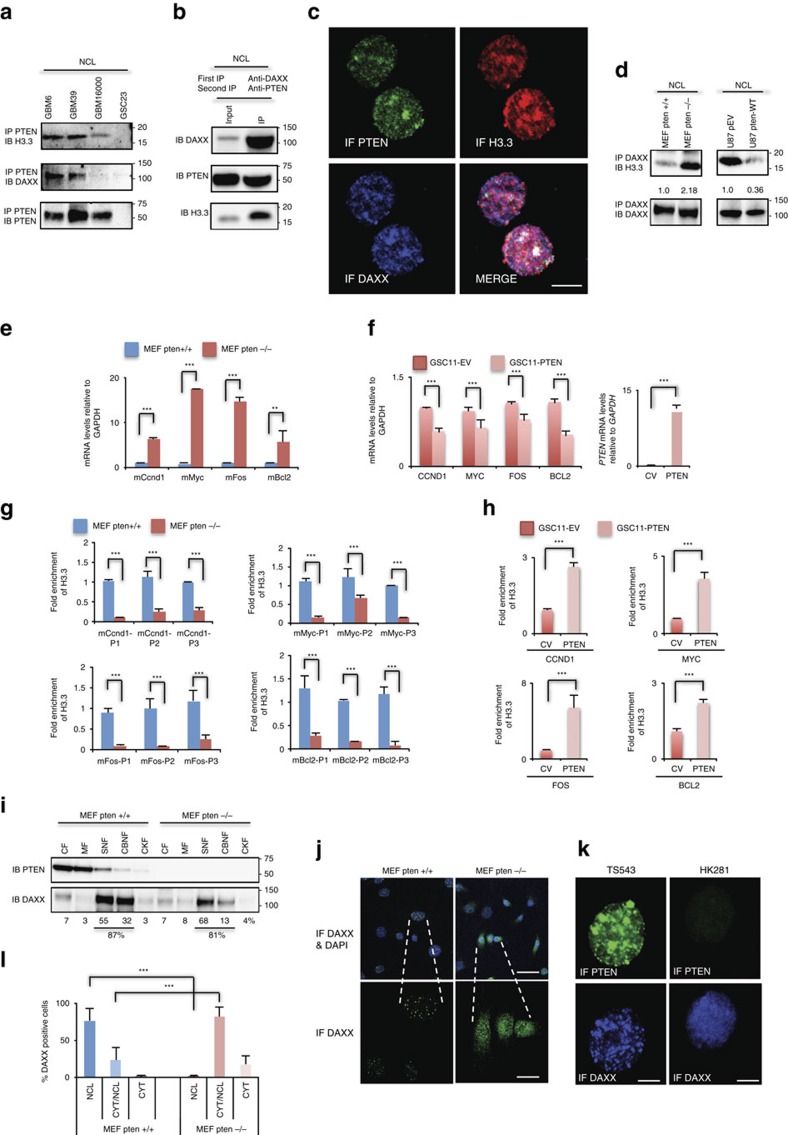
PTEN represses oncogene expression by controlling H3.3 deposition on chromatin and H3.3-DAXX interaction. (**a**) Co-immunoprecipitation analysis of anti-PTEN followed by anti-H3.3 or anti-DAXX immunoblotting were performed using nuclear protein (NCL) from patient-derived glioma spheres. (**b**) Sequential immunoprecipitation from nuclear extracts of HK281-PTEN GBM-spheres. First IP anti-DAXX and second IP anti-PTEN, and immunoblots anti-DAXX, anti-PTEN and anti-H3.3. (**c**) Confocal immunofluorescence analysis of endogenous PTEN, H3.3 and DAXX proteins in TS576 GBM-spheres (Pearson’s coefficient in colocalized region equal to 0.4168). Scale bar, 5 μm. (**d**) Nuclear proteins from MEF pten+/+ and MEF pten−/− (left), and U87 glioma cells (right) transfected with empty vector pEV or PTEN-WT were immunoprecipitated with anti-DAXX followed by immunoblotting with anti-H3.3. (**e**,**f**) mRNA expression analysis of *CCND1*, *MYC*, *FOS* and *BCL2* by RT–qPCR in *PTEN-WT or PTEN-*deficient cells (*n*=3 biological samples with three replicates each, **P<0.001, ***P<0.0001, one-way ANOVA). (**g**,**h**) ChIP-qPCR of H3.3 in *PTEN-WT or PTEN-*deficient cell, using different sets of primers that anneal within 1–2 Kb of the transcription start site. For ChIP assays, bar graphs indicate fold enrichment of H3.3 over input (*n*=3 biological samples with three replicates each, ***P<0.0001, one-way ANOVA). (**i**) Cellular fractionation was carried out in MEF cells followed by anti-DAXX and anti-PTEN immunoblotting. DAXX expression was quantified by densitometry analysis. (**j**,**k**) Representative immunofluorescence images of endogenous DAXX expression in MEFs (**j**) or GBM-spheres (**k**). For **j** top scale bar is 20 μm and bottom scale bar is 50 μm. For **k** scale bar is 5 μm. (**l**) Percentage of DAXX-positive cells in different cellular compartments in *Pten-wt and Pten-null* MEFs. NCL: nuclear; CYT/NCL: cytosolic and nuclear; CYT: cytosolic; NS: no significant differences. Error bars represent s.e.m. from three different experiments, ***P<0.0001, one-way ANOVA.

DAXX is a histone chaperone protein that directly interacts with the histone H3.3 variant and facilitates its deposition on chromatin^23^,^24^,^38^. Using protein lysates from patient-derived glioma neurospheres (GBM-PDX) that express endogenous PTEN, DAXX and H3.3, we examined whether the PTEN-DAXX complex identified above interacts with H3.3. As shown in Fig. 2a and Supplementary Fig. 1a,b, endogenous PTEN co-immunoprecipitated with H3.3 and DAXX in GBM-sphere samples. We corroborated this result by performing sequential immunoprecipitation from nuclear extracts of a PTEN-null glioma-sphere that over-expresses exogenous PTEN (HK281-PTEN) (Fig. 2b), and also by quantitative immunofluorescence of endogenous proteins in GBM-spheres (Fig. 2c and Supplementary Fig. 1c,d Pearson’s coefficient in co-localized region equal to 0.4168 for TS576, 0.4526 for GBM39, 0.7665 for TS543 and 0.7344 for TS528 GBM-spheres). These results confirm the presence of a nuclear PTEN–DAXX–H3.3 tripartite complex in patient-derived GBM neurospheres.

We then asked if the PTEN–DAXX complex modulated the histone H3.3 chaperone function of DAXX. By immunonprecipitating DAXX from nuclear protein extracts of *Pten-wt* and *Pten-null* MEF (mouse embryonic fibroblast) cells we observed that the levels of H3.3 that co-immunoprecipitated with DAXX increased twofold in *Pten-null* cells when compared with *Pten-wt* cells (Fig. 2d, left panel and Supplementary Fig. 1e). Conversely, upon reconstitution of *PTEN* expression in a *PTEN*-deficient glioma cell line (U87), we observed a threefold reduction in the levels of H3.3 bound to DAXX (Fig. 2d, right panels and Supplementary Fig. 1e). These results indicate that DAXX–H3.3 interaction could be regulated by *PTEN* expression.

H3.3 deposition has been associated with both active and repressive chromatin^12^,^13^, while DAXX has been associated exclusively with gene repression^35^,^36^. This dichotomy led us to investigate the role of PTEN in the regulation of H3.3 deposition on chromatin and consequent gene expression. Four genes, either involved in neural stem cell proliferation and/or neuronal activity that have been reported to be regulated by PTEN^39^,^40^,^41^,^42^,^43^ and DAXX^35^,^38^, were analysed (*CCND1*, *MYC*, *FOS* and *BCL2*). Both RT–qPCR and anti-H3.3 chromatin immunoprecipitation (ChIP) were performed in *PTEN-NULL* and *PTEN-WT* MEFs, glioma cells and GBM-spheres. These experiments showed an inverse correlation between gene expression (Fig. 2e,f and Supplementary Fig. 2a,b) and H3.3 enrichment (Fig. 2g,h and Supplementary Fig. 2c) in *PTEN-*deficient cells compared with *PTEN-WT* cells (*P*<0.0001), suggesting that PTEN represses oncogene expression by recruiting H3.3 to chromatin. These data were corroborated by performing ChIP assays for other markers of active (RNA pol-II) and repressive (H3K27me3) chromatin, which showed a strong correlation between high H3.3 levels and a high H3K27me3 signal in the presence of PTEN expression (Supplementary Fig. 2d, *P*<0.0001); and an inverse correlation between low H3.3 levels and a high RNA Pol-II signal in *PTEN-*deficient cells (Supplementary Fig. 2e, *P*<0.001). Taken together, these data show that *PTEN* represses gene expression by increasing the levels of H3.3 bound to chromatin.

Regulation of gene expression by PTEN has been reported previously^9^. To determine if changes in the amount of H3.3 bound to chromatin were dependent on the lipid phosphatase activity of PTEN, ChIP assays were performed with anti-H3.3 in *PTEN*-deficient cells reconstituted with PTEN-wild type (PTEN-WT) or a PTEN-lipid and protein phosphatase inactive mutant (PTEN-G129R). As shown in Supplementary Fig. 2f,g, PTEN-G129R increased the amount of H3.3 bound to chromatin to the same level as PTEN-WT and also reduced the levels of H3.3 bound to DAXX (Supplementary Fig. 2h), indicating that H3.3 deposition on chromatin was regulated by PTEN independently of its phosphatase activity.

DAXX has been shown to compete with DNA for H3.3-H4 tetramer formation^44^,^45^ and we found that PTEN competes with H3.3 for the DAXX-histone binding domain (Fig. 1e). Based on these results, the effect of PTEN on DAXX cellular compartmentalization was investigated using cell fractionation. As shown in Fig. 2i, a reduction in the DAXX-chromatin fraction and a reciprocal increase in the DAXX soluble nuclear fraction occurred in *Pten-null* cells compared with *Pten-wt* cells. Upon *PTEN* reconstitution in *PTEN*-deficient cells, the amount of DAXX associated with chromatin increased from 37 to 61% (Supplementary Fig. 3a). Corroborating these results, DAXX immunofluorescence quantification in *PTEN-WT* cells displayed a specific signal associated with nuclear bodies (Fig. 2j–l and Supplementary Fig. 3b); however, the DAXX signal was highly diffuse in the nucleus and cytosol in *PTEN*-deficient cells (Fig. 2j–l and Supplementary Fig. 3b). No changes in nuclear body distribution of ATRX and HIRA were observed in *Pten-wt* and *Pten-null* cells (Supplementary Fig. 3c,d). In order to interrogate if ATRX or an ATRX mutant (R1426*) that is present in gliomas affect DAXX-PTEN and/or DAXX-H3.3 association we overexpressed these constructs and analysed their effect. No changes in DAXX–PTEN and DAXX–H3.3 associations were observed after overexpressing ATRX-WT or ATRX-R1426* mutant (Supplementary Fig. 3e). Taken together, these results show that PTEN regulates oncogene expression by controlling DAXX–H3.3 association and deposition on chromatin independently of ATRX.

### *DAXX* suppression compromises *PTEN*-deficient cells

**Figure 3:**
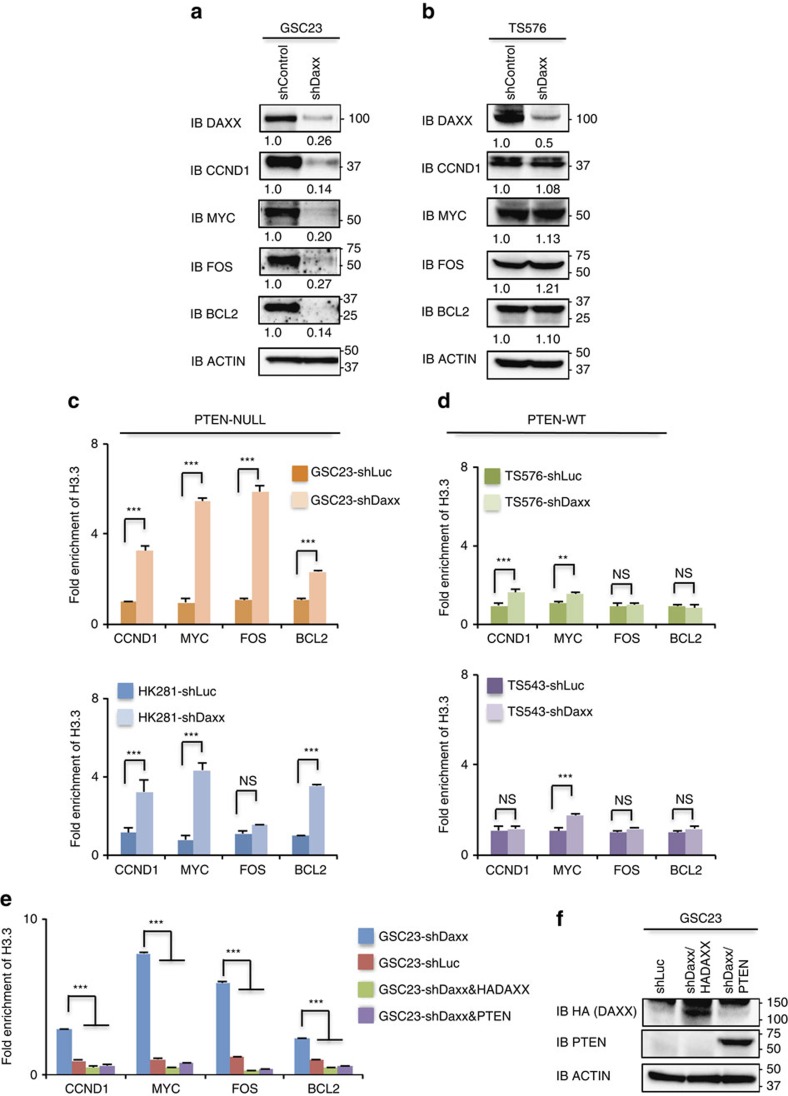
*DAXX* disruption compromises gene expression and restores H3.3 deposition in *PTEN*-deficient cells. (**a**,**b**) Total proteins were analysed by western blot in *PTEN-*deficient (**a**) or *PTEN-*expressing (**b**) GBM-spheres transduced with lentivirus shRNA control (shLuc) or shRNAs targeting *DAXX* (shDaxx). Actin is a loading control. (**c**,**d**) anti-H3.3 ChIP-qPCR was performed in *PTEN-*deficient (**c**) or *PTEN-WT* (**d**) GBM-spheres with stable knockdown of DAXX or with shControl. (**e**) ChIP-qPCR of H3.3 in Daxx-kd/PTEN-deficient GBM-spheres expressing a HA-DAXX-shRNA-resistant vector or PTEN-WT. For ChIP assays, bar graphs indicate fold enrichment of H3.3 over input (*n*=3 biological samples with three replicates each, **P<0.001, ***P<0.0001, one-way ANOVA). (**f**) Immunoblot analysis of HA-DAXX and PTEN expression in DAXX-kd or shControl GSC23 neurospheres. NS: no significant differences. Error bars represent s.e.m. from three different experiments. Numbers under the blots indicate fold ratios of protein levels relative to shControl after normalization to actin.

To investigate if *DAXX* disruption can restore H3.3 deposition to the oncogene promoters interrogated in Fig. 2, and consequently suppresses their expression in *PTEN*-deficient cells, lentiviral-encoded shRNAs targeting *DAXX* were used to generate stable DAXX-knockdown (Daxx-kd) in glioma cell lines and GBM-PDX neurospheres. Upon immunoblot analysis, DAXX-kd cells showed a decrease in CYCLIN-D1, MYC, FOS and BCL2 protein expression when compared to shControl cells (Fig. 3a and Supplementary Figs 4a and 5a). In concordance with these results, H3.3 enrichment on the promoters of *CCND1*, *MYC*, *FOS* and *BCL2* was observed after *DAXX* knockdown in *PTEN*-deficient cells (*P*<0.001) compared with shControl cells (Fig. 3c and Supplementary Figs 4b and 5b). By performing a promoter reporter assay we corroborated a direct repression of *CYCLIND1* and *MYC* promoter activity in DAXX-kd/PTEN-null cells compared with shControl cells (Supplementary Fig. 4c), thus confirming direct DAXX-mediated regulation of these genes in *PTEN*-deficient cells.

Because MYC and CYCLIN-D1 play important roles in regulating cell cycle progression and proliferation^46^,^47^, the effect of *DAXX* inhibition on these cellular functions was analysed in *PTEN*-deficient cells. These experiments showed that a greater percentage of cells were arrested in the G1 and G2 phases of the cell cycle in DAXX-kd/PTEN-null cells (15 and 20%, *P*<0.001) compared to shControl cells (Supplementary Figs 4d and 5c). Knocking down *DAXX* expression in *PTEN*-deficient cells also suppressed cell proliferation (*P*<0.001), while shControl/PTEN-null cells proliferated 2.5-fold faster than DAXX-kd/PTEN-null cells (Supplementary Figs 4e and 5d). No changes in cell cycle progression were seen in any of the GBM-spheres cells after *DAXX-kd* (Supplementary Fig. 5e), explained in part by their quiescent or slowly proliferative nature^48^,^49^. Together, these results illustrate that *DAXX* inhibition in *PTEN-*deficient cells restores the deposition of H3.3 on chromatin, promotes oncogene repression and compromises cellular proliferation.

In order to confirm that DAXX-knockdown directly affects oncogene expression through H3.3 and to eliminate the possibility of off-target effects, we reconstituted DAXX-kd/PTEN-deficient cells with a DAXX-shRNA-resistant construct (HA-DAXX). As shown in Supplementary Fig. 6a,b protein and mRNA levels of *CCND1*, *MYC*, *FOS* and *BCL2* genes were restored after DAXX re-expression in DAXX-kd cells. H3.3 enrichment on the promoter of those genes was also restored to normal levels compared with shControl cells after reconstitution of HA-DAXX or PTEN re-expression in DAXX-kd/PTEN-deficient cells (Fig. 3e,f and Supplementary Fig. 6c), as well as cellular proliferation (Supplementary Fig. 6d) and oncogene promoter activity (Supplementary Fig. 6e,f); confirming that DAXX was responsible for these cellular functions.

To test if these cellular and molecular effects observed after *DAXX* knockdown were *PTEN* dependent, *DAXX* expression was knocked down in glioma cell lines and GBM-spheres that express wild-type *PTEN*. No significant changes in total protein levels of CYCLIN-D1, MYC, FOS and BCL2 were detected after *DAXX* knockdown in *PTEN-WT* cells compared with shControl/PTEN-WT cells (Fig. 3b and Supplementary Fig. 7a,e). Additionally, no or faint changes in the enrichment of H3.3 (Fig. 3d and Supplementary Fig. 7b) or in cell cycle progression (Supplementary Fig. 7c,f) were observed in Daxx-kd/PTEN-WT cells in comparison with shControl cells. DAXX knockdown in *PTEN-WT* cells slightly affected cell proliferation in glioma cells (Supplementary Fig. 7d,g), which is perhaps related to other molecular mechanisms associated with DAXX^50^. These data indicate that PTEN is a critical component for H3.3 chromatin deposition-associated gene repression, while *DAXX* suppression, in the context of *PTEN* deficiency, re-establishes H3.3-mediated oncogene repression (Fig. 6h and Supplementary Fig. 21).

Similarly, we observed a decrease in H3.3 loading on oncogene promoters (Supplementary Fig. 8a) and an increase in oncogene expression (Supplementary Fig. 8b) in *Daxx*-null MEF cells (MEF Daxx −/−) compared with *Daxx*-expressing cells (MEF Daxx +/+). Furthermore, *Pten* inhibition in *Daxx*-deficient cells (MEF Daxx −/−shPten, Supplementary Fig. 8c) restored H3.3 enrichment on the chromatin (Supplementary Fig. 8d) and repressed oncogene expression (Supplementary Fig. 8e), blocking cell proliferation of *Daxx*-deficient cells (Supplementary Fig. 8f,g). We speculate that oncogene expression is driven in *Daxx*-deficient MEFs in part because Pten still binds to H3.3 in the absence of *Daxx* expression (Supplementary Fig. 8h).

### *DAXX* inhibition affects *PTEN*-null glioma spheres

**Figure 4:**
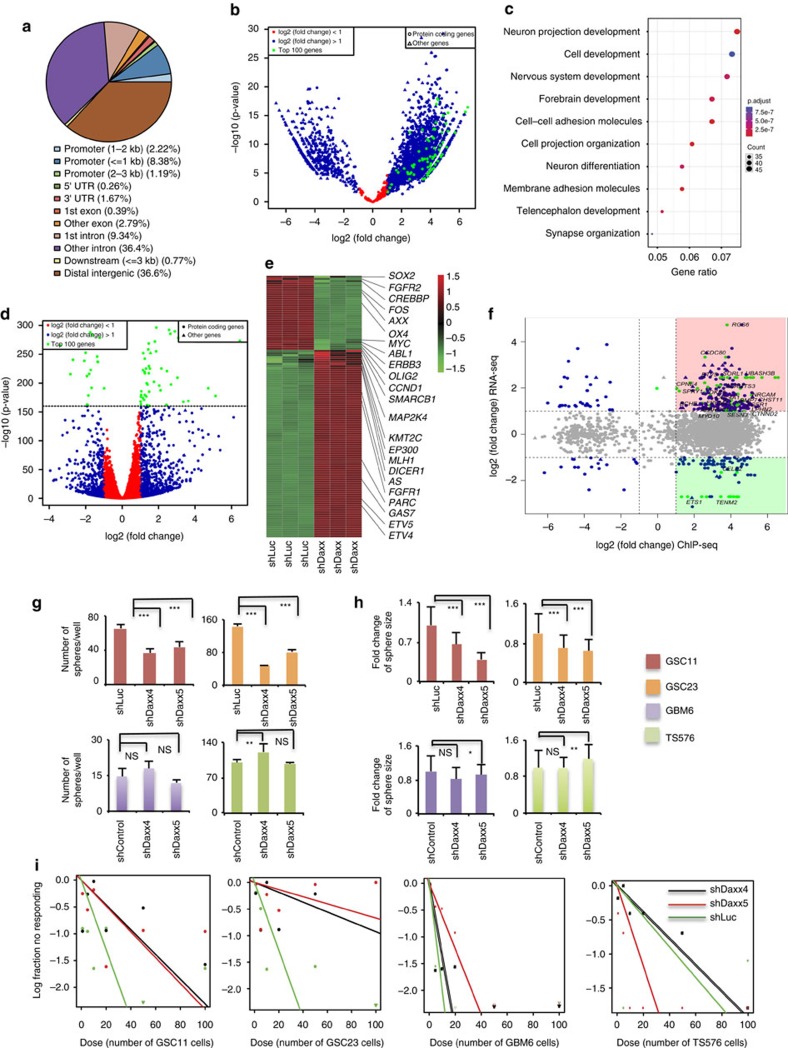
*DAXX* inhibition affects H3.3 genomic enrichment, gene expression and oncogenesis in *PTEN-null*/GBM cells. (**a**) Genomic distribution of H3.3 peaks in *DAXX* knockdown GBM cells. (**b**) Volcano plot of H3.3 differential binding (DiffBind) peaks between shControl (shLuc) and shDaxx *PTEN-null*/GBM samples by ChIP-seq (*P*<0.05&log2(Fold Change)>1). (**c**) Gene ontology analysis of H3.3 differential binding genes in *DAXX* knockdown *PTEN-*deficient GBM-spheres. (**d**) Volcano plot of differentially expressed (DiffExp) gene between shLuc and shDaxx *PTEN-null*/GBM samples by RNA-seq (*P*<0.05&log2(Fold Change)>1). (**e**) Heatmap of Top 100 DiffExp genes highlighting tumour suppressors genes and oncogenes according to the Cancer Gene Census in DAXX-kd compared with shControl (shLuc) cells (*P*-value<0.05, log2(Fold change)>1). (**f**) Scatter plot of overlay genes between H3.3 DiffBind and DiffExp genes from ChIP- and RNA-seq, respectively. Red square, upregulated genes with H3.3 enrichment. Green square, downregulated genes with H3.3 enrichment (*P*<0.05&log2(Fold Change)>1). Top 100 DiffExp genes are labelled with green dots in ChIP- and RNA-seq plots and scatter plot. (**g**,**h**) Quantification of the total number of spheres (**g**) and sphere size (**h**) in GBM-PDX lines transduced with shLuc or shDaxx (*n*=3 biological samples with six replicates each, NS: no significant differences, *P<0.05, **P<0.001 and ***P<0.0001, one-way ANOVA). (**i**) Plots of sphere-forming frequencies using *PTEN-NULL* (GSC11 and GSC23) and *PTEN-WT* (GBM6 and TS576) GBM-PDX neurospheres, after stable knockdown of *DAXX* or shControl. The assay was performed by *in vitro* limiting dilution using a 0.95 confidence interval.

To further identify genome-wide H3.3 distribution changes after *DAXX* inhibition in *PTEN-null*/GBM-PDX samples, we performed unbiased chromatin immunoprecipitation sequencing (ChIP-seq) (Supplementary Fig. 9a). We observed that most of the H3.3 enrichment in *DAXX* knockdown cells occurred in distal intergenic (37%) and intronic (36%) regions and about 10% in promoter regions (Fig. 4a). Of the 3,200 peaks displaying a H3.3 differential binding pattern (DiffBind), there were 1,425 genes with a positive fold change (FC) and 326 genes with a negative FC (*P*<0.05 & log2 (Fold Change)>1, Fig. 4b). Gene ontology and pathway analysis of H3.3 differentially enriched genes correlated with pathways associated with metabolic process, nervous system development, neuronal differentiation and the cell cycle (Fig. 4c and Supplementary Fig. 9b,c). Furthermore, to examine transcriptome changes in *DAXX-kd*/*PTEN-null* GBM cells we performed RNA sequencing (RNA-seq) (Supplementary Fig. 11a). Of the 1,403 genes showing differential expression (DiffExp) between cells treated with shControl and cells treated with shDAXX, there were 846 upregulated genes and 557 downregulated genes (*P*<0.05 and log2(FoldChange)>1, Fig. 4d). *DAXX*-kd resulted in a significant upregulation of several tumour suppressor genes and downregulation of various oncogenes, including *CCND1*, *MYC*, *FOS*, *SOX2* and *OLIG2* (Fig. 4e and Supplementary Fig. 10). Additionally, the most significant pathways were neuronal differentiation, nervous system development, neurogenesis and regulation of cell differentiation (Supplementary Fig. 11b).

Further analyses were performed overlapping the H3.3 DiffBind peaks from ChIP-seq with DiffExp genes from RNA-seq. In total 1,390 genes were overlapping between the two data sets, where a subset of 133 genes were upregulated and enriched for H3.3, and 37 genes were downregulated and enriched for H3.3 (*P*<0.05 and log2foldChange>1) (Fig. 4f). Gene ontology analysis confirmed enrichment in pathways associated with nervous system development, regulation of MAPK cascade and neuronal differentiation (Supplementary Fig. 11c,d). These data show that *DAXX* inhibition robustly alters H3.3 genomic distribution leading to affects on gene expression in *PTEN-null*/GBM neurospheres.

We next studied whether *DAXX* inhibition can compromise the oncogenic behaviour of GBM-PDX cells grown in neurosphere conditions (Supplementary Fig. 12a). Immunoblot analysis of transcription factors involved in the development of gliomas (OLIG2, MYC, SOX2 and PAX6)^51^,^52^,^53^ showed a specific decrease in expression in *PTEN-*deficient cells (GSC11, GSC23, HK281) compared with *PTEN-WT* cells (GBM6, TS675, GBM39, TS543) (Supplementary Fig. 12b,c), suggesting that *DAXX* disruption in *PTEN-null*/GBM-PDXs affects the expression of transcription factors implicated in gliomagenesis. A downregulation of ATRX expression was also observed after *DAXX* inhibition in both conditions (Supplementary Fig. 12c), PTEN-expressing and PTEN-null GBM-spheres, as has previously been reported by other groups^12^,^23^.

Additionally, we determined if *DAXX* knockdown affects GBM-PDX proliferation. Knocking down *DAXX* resulted in a specific reduction of number and size of spheres formed by *PTEN-null* (GSC11 and GSC23) cells, compared with *PTEN*-expressing (GBM6 and TS576) cells (Fig. 4g,h), *P*<0.001. Furthermore, using an *in vitro* limiting dilution assay, *DAXX* knockdown resulted in a three- to eightfold reduction in the self-renewal capacity of *PTEN-null* GBM-PDXs compared with DAXX-kd/*PTEN-WT* spheres (Fig. 4i, Supplementary Fig. 13).

In order to investigate if DNA replication was affected after DAXX disruption, since it has been reported that PTEN regulates DNA regulation and repair^9^,^54^, we quantified the percentage of positive cells relative to two components of the fork replication complex (RPA32-P and ATRIP-P). As is shown in Supplementary Fig. 14 *DAXX* inhibition increases the percentage of RPA32-P- and ATRIP-P-positive cells (GSC23, 17% and 12% increase, respectively) specifically in *PTEN*-null GBM-spheres. We next evaluated the effect of *DAXX* suppression on the differentiation capacity of GBM-PDX neurospheres. In contrast to shControl cells, there was a 30–50% decrease in OLIG2 (Supplementary Fig. 15a, *P*<0.0010.0001) and a 20–30% decrease in GFAP-positive cells (Supplementary Fig. 15b, *P*<0.001) when *DAXX* knockdown cells were incubated in differentiation conditions. In general, these results illustrate that *DAXX* inhibition, independent of ATRX, disrupts GBM-PDX oncogenic properties selectively in a *PTEN-*deficient genetic background, in part through downregulation of transcription factors that preserve glioma proliferation and upregulation of tumour suppressor genes.

### *DAXX* inhibition suppresses tumor growth and improves survival

**Figure 5:**
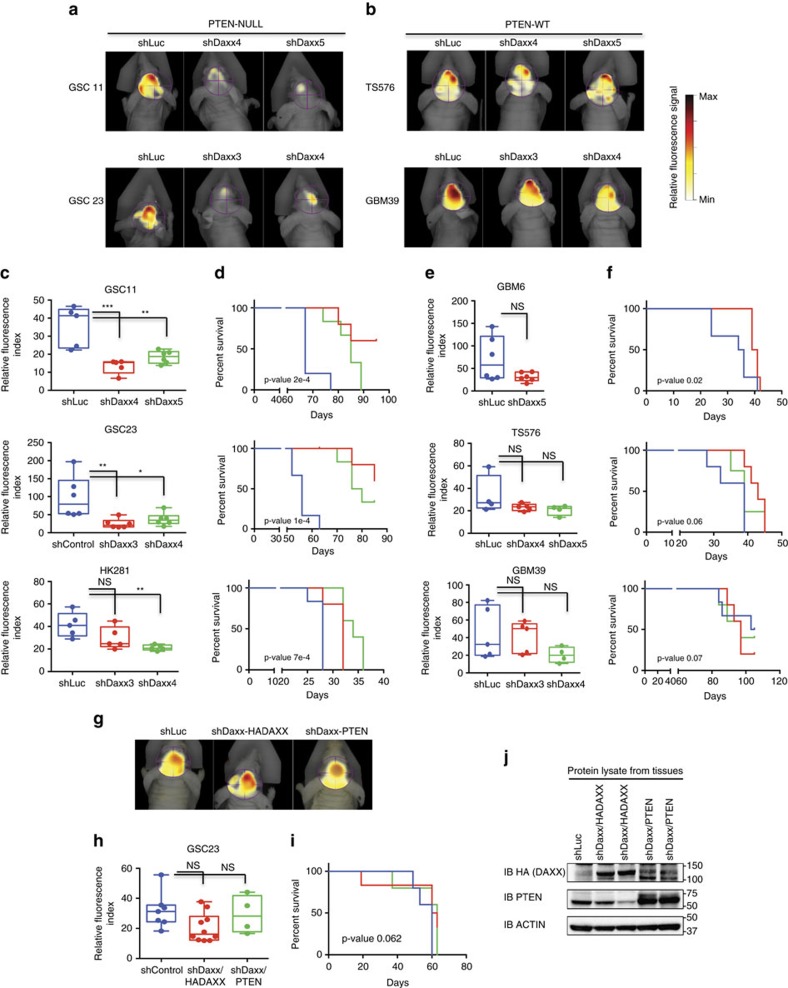
DAXX disruption inhibits intracranial tumour growth and improves survival rate in PTEN-deficient GBM-PDX models. (**a**,**b**) Representative fluorescence molecular tomography (FMT) images of mice intracranially engrafted with *PTEN*-deficient (**a**) or *PTEN*-expressing (**b**) GBM-PDX neurospheres. Glioma spheres were co-transduced with lentivirus-encoded shRNAs targeting *DAXX* (shDaxx) or shRNA Control (shLuc), and a near infrared fluorescence protein. Relative fluorescence signal was monitored by FMT. (**c**,**e**) Relative fluorescence quantification of GSC11, GSC23 and HK281 *PTEN-NULL* (**c**), and GBM6, TS765 and GBM39 *PTEN-WT* (**e**) glioma-PDX xenografts analysed by FMT imaging (*P<0.05, **P<0.001 and ***P<0.0001, one-way ANOVA). (**d**,**f**) Kaplan–Meier survival curves of mice implanted with *PTEN*-deficient (**d**) or wild-type *PTEN* (**f**) GBM-PDXs expressing shDaxx or shControl. (**g**–**j**) *In vivo* rescue experiments in DAXX-kd/PTEN-deficient GSC23 engrafted mice after re-expression of HA-DAXX-shRNA-resistant (shDaxx/HADAXX) or PTEN-WT (shDaxx/PTEN). (**g**) Representative FMT images, (**h**) Relative fluorescence quantification, (**i**) Kaplan–Meier survival curves and (**j**) immunoblot analysis from tissues. PTEN antibody also detects endogenous mouse-Pten in shLuc and shDaxx samples. NS: no significant differences.

To assess whether *DAXX-*knockdown affects intracranial tumour growth, we infected glioma-PDX neurospheres with a lentivirus-encoded shRNA targeting *DAXX* (shDaxx) or control shRNA (shLuc), along with a lentivirus expressing a near-infrared fluorescent protein. Imaging using fluorescence molecular tomography showed a decrease in fluorescence signal in mice engrafted with shDAXX/*PTEN-*deficient (GSC11, GSC23, HK281) GBM neurospheres in comparison with animals implanted with shDAXX/*PTEN-WT* (TS576, GBM39, GBM6, TS543) GBM cells (Fig. 5a,b Supplementary Fig. 16c,d). Relative fluorescence quantification showed a statistically significant (*P*<0.0001) reduction in tumour growth for DAXX-kd/*PTEN-*deficient glioma xenografts (Fig. 5c, Supplementary Fig. 16a,e) compared with mice implanted with DAXX-kd/*PTEN-WT* GBM spheres (Fig. 5e, Supplementary Fig. 16b,e). Moreover, survival analysis showed mice intracranially engrafted with DAXX-kd/*PTEN-*deficient GBM cells (Fig. 5d, Supplementary Fig. 16f) survived significantly (*P*<0.0001) longer than animals implanted with DAXX-kd/*PTEN-WT* GBM spheres (Fig. 5f and Supplementary Fig. 16f).

We validated that *DAXX* inhibition directly affects tumour size and survival of PTEN-null glioma xenografts by restoring DAXX or PTEN expression in DAXX-kd/PTEN-deficient engrafted mice. Neither changes in fluorescence signal nor significant differences in tumour size and survival were observed in DAXX-kd/PTEN-null xenografts (Fig. 5g–i, Supplementary Fig. 16g) after re-expression of HA-DAXX or PTEN-WT (Fig. 5j). Histological analyses of mouse brain tumours showed angiogenesis, haemorrhagic areas, necrosis and high mitotic activity in tumours derived from shLuc and shDAXX/PTEN-WT GBM-spheres (TS576; Supplementary Fig 17a,b,g). In contrast, no haemorrhagic areas, angiogenesis, or necrosis, and low mitosis were observed in brains implanted with shDAXX/PTEN-null GBM-spheres compared to shLuc/PTEN-null-spheres (GSC23; Supplementary Fig. 17c,d,g). These histological features were observed after re-expression of DAXX and PTEN in animals bearing shDAXX/PTEN-null GBM-spheres (GSC23; Supplementary Fig. 17e,f,g). Our studies indicate that *DAXX* suppression inhibits GBM-PDX tumour growth and extends overall survival specifically in mice engrafted with *PTEN-*deficient GBM cells.

### *DAXX* expression is upregulated in gliomas

**Figure 6:**
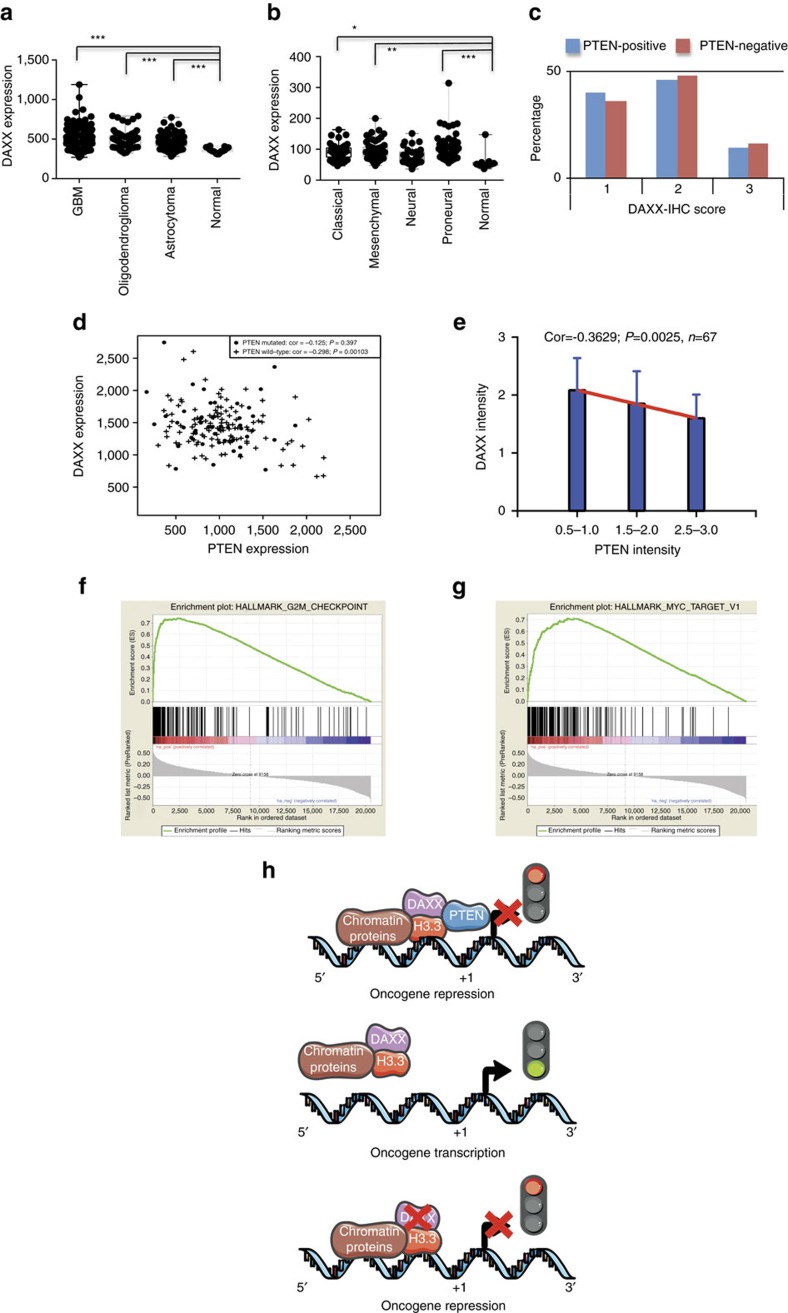
DAXX expression is altered in GBMs. (**a**) REMBRANDT data analysis of *DAXX* expression in gliomas compared with normal brain. (**b**) TCGA data analysis of *DAXX* expression in GBM subtypes (*P<0.05, **P<0.001 and ***P<0.0001, one-way ANOVA). (**c**) Immunohistochemistry analysis of DAXX expression signal in PTEN-positive and PTEN-negative adult GBM tissues (Chi-square 0.7639, *P*=0.6825, *n*=68). (**d**) Anti-correlated expression of *DAXX* and *PTEN* in GBMs from the TCGA database. (**e**) Immunohistochemistry quantification of DAXX and PTEN intensity signal from adult GBM tissues (cor=−0.3721 *P*=0.0025, *n*=67). (**f**,**g**) Gene set enrichment analysis (GSEA) showing the most overrepresented gene sets among positively correlated: (**f**) G2M checkpoint and (**g**) MYC targets (enrichment score=0.744 and 0.713; FDR q-value=0.000; FWER *P* value=0.000). (**h**) Schematic diagram showing that in the presence of PTEN (top sketch) oncogene transcription is repressed by a PTEN-DAXX-H3.3 chromatin complex in association with other chromatin proteins. Conversely, upon loss of PTEN (middle sketch) oncogene transcription is activated. DAXX inhibition in the context of PTEN deficiency (bottom sketch) restores H3.3 on chromatin and hence represses oncogene transcription.

Having established that *DAXX* disruption inhibits tumour growth and increases survival in GBM-PDX models, we next examined if *DAXX* gene expression was altered in different human gliomas. By using the REMBRANDT and TCGA databases we found a statistically significant (*P*<0.0001) upregulation of *DAXX* expression in GBMs, oligodendrogliomas and astrocytomas in comparison with normal brain (Fig. 6a). This was apparent in the classical, mesenchymal and proneural GBM subtypes (Fig. 6b). However, no significant changes in DAXX protein signal were observed within PTEN-positive and PTEN-negative adult GBM samples (Fig. 6c, Chi-square 0.7639, *P*=0.6825, *n*=68); but a significant anticorrelated expression (cor=−0.298, *P*=0.001, *n*=166) between *DAXX* and *PTEN-WT* was found in the GBM-TCGA database (Fig. 6d) and confirmed by immunohistochemistry in adult GBM tissues (Fig. 6e, cor=−0.3721 *P*=0.0025, *n*=67). A similar anticorrelated *DAXX*/*PTEN* expression pattern was also observed in invasive breast carcinoma (BRCA, cor=−0.325, *P*=1.26e-27, *n*=1,096) from the TCGA data set (Supplementary Fig. 18a); suggesting a more general cancer-related gene expression regulatory mechanism. No significant correlation was observed between *ATRX/PTEN* and *HIRA/PTEN* (Supplementary Fig. 18b,c), indicating a specific PTEN–DAXX regulatory mechanism.

We next investigated the biological consequence of *DAXX* upregulation in human GBMs. Gene set enrichment analysis showed that the three most overrepresented gene sets that positively correlated with *DAXX* expression in GBM patients were E2F targets, G2M checkpoint components and MYC targets (Fig. 6f,g); in concordance with our data showing that *DAXX* knockdown affects cell cycle progression, cellular proliferation (Supplementary Figs 4 and 5) and tumour growth (Fig. 5 and Supplementary Fig. 16). The prognostic impact of *DAXX* genetic alterations (mutations) in GBMs was also interrogated using the TCGA data set. We found that 1% of GBMs have *DAXX* alterations (missense mutations); however, a difference in overall survival rate compared with non-altered cases was not apparent (Supplementary Fig. 19a, *P* value 0.544). The overall survival rate in patients with both *DAXX* and *PTEN* alterations was not significant (*P* value 0.976) in GBM cases (Supplementary Fig. 19b). Our analysis indicates that *DAXX* expression is upregulated in gliomas and inversely correlated with *PTEN*.

Finally, we analysed gene expression levels in the normal human brain using the Allen human brain database^55^. Here, it was determined that *PTEN* and *H3F3B* (H3.3) have similar expression profiles in comparison with *CCND1*, *MYC*, *FOS* and *BCL2* genes in the same brain structure regions (Supplementary Fig. 20). Particularly in the metencephalon (MET), the intensity of *PTEN* expression was 3–6 times higher than expression of the other analysed genes, using different probes. These analyses are consistent with our general model, proposing that PTEN controls gene expression through the regulation of H3.3 and DAXX.

## Discussion

Our study suggests that PTEN is part of a chromatin complex with DAXX and H3.3, and negatively regulates genes involved in oncogenesis (Fig. 6h and Supplementary Fig. 21). Since DAXX recruits proteins like H3.3 to PML-nuclear bodies (PML-NBs)^24^,^56^, and PML-NBs have been shown to regulate PTEN^37^, we propose that DAXX, H3.3, PML and PTEN may form a chromatin complex that regulates gene transcription. We suggest that in the absence of *PTEN* an unincorporated H3.3-chromatin fraction is recruited to PML-NBs in a DAXX-dependent manner^56^, leading to an increase in a DAXX–H3.3 soluble fraction. Upon *DAXX* inhibition, we speculate that H3.3 is liberated from PML-NBs and is hence restored for chromatin binding.

We propose a model that in *PTEN*-deficient tumour cells, DAXX removes H3.3 from chromatin (Fig. 6h, Supplementary Fig. 21a), probably by competing for chromatin binding, as has been reported by other groups^45^. Therefore, inhibition of *DAXX* restores H3.3 on the chromatin and inhibits oncogene expression (Fig. 6h, Supplementary Fig. 21b). The anti-tumorigenic effect mediated by DAXX inhibition does not work in PTEN-expressing cells because PTEN can also bind to H3.3 and we speculate that this attenuates H3.3 chromatin binding (Supplementary Fig. 21c).

Four tumour-related genes associated with neural stem cell proliferation and neuronal activity, and regulated by PTEN^39^,^40^,^41^,^42^,^43^ and DAXX^35^,^38^ were studied. Particularly, *MYC* and *CCND1* have been reported to be upregulated after *PTEN* disruption in progenitor cells^40^,^42^, and associated with brain hyper-proliferation in a *Pten* knockout mouse model^40^. Here we demonstrate that PTEN impinges upon *MYC* and *CCND1* expression at the transcriptional level by increasing the loading of a repressive DAXX–H3.3 complex on the chromatin. In contrast, *MYC* and *CCND1* overexpression that occurs in the context of *PTEN* deficiency can be abrogated by *DAXX* inhibition, which restores chromatin loading of repressive H3.3. Furthermore, genomic analysis in *DAXX*-knockdown/*PTEN*-deficient GBM samples display a genome-wide H3.3 distribution change (1,751 genes with H3.3 different binding signal) and upregulation of several tumour suppressor genes and downregulation of various oncogenes (1,403 genes with differential expression), including *CCND1*, *MYC*, *FOS*, *SOX2* and *OLIG2,* compared with shControl/*PTEN*-deficient GBM samples. In concordance with the literature^12^,^38^,^57^, we show that H3.3 enrichment correlates with an upregulation and downregulation of genes involved in nervous system development and neuronal differentiation.

It is well documented that the H3.3 variant is regulated and incorporated in the chromatin by distinct proteins. Histone H3.3 is preferentially loaded at euchromatic regions by HIRA^12^,^22^ and at heterochromatic regions by DAXX^23^,^24^. Furthermore, the ATRX/DAXX complex is required for targeting H3.3 to telomeric chromatin^12^. However, DAXX and ATRX have a distinct chromatin-binding profile, where DAXX preferentially binds to promoter regions^58^ and regulates H3.3 loading of immediate early genes after neuronal stimulation^38^. More recently, it was reported that MLL5 (Mixed Lineage Leukemia 5) represses H3.3 expression in adult GBMs allowing global reorganization of chromatin and self-renewal^20^. These data suggest that several histone chaperons and chromatin regulator proteins, including PTEN, can be involved in H3.3 deposition and its expression, and consequently chromatin regulation. In this study, we did not observe changes in the expression or localization of ATRX and HIRA in *PTEN-*deficient cells, no changes in DAXX–PTEN and DAXX–H3.3 association after ATRX overexpression, nor a correlated expression with *PTEN-WT.* However, we did detect a downregulation of expression of ATRX after DAXX inhibition, as has been reported previously^12^,^23^. Our results indicate that DAXX disruption specifically affects GBM-PDX oncogenesis in *PTEN*-null models independently of ATRX.

*DAXX* and *ATRX* mutations have also been correlated with an alternative lengthening of telomeres (ALT) phenotype in pancreatic neuroendocrine tumours^59^ and paediatric GBMs^14^; however, intact telomeres have been observed in *PTEN*-deficient cells^9^, where chromosomal translocations and centromeres breakages are mainly affected. We reason that because genetic alterations of *DAXX* are uncommon in adult GBMs, oncogene transcription and chromosomal instability may drive cellular transformation mediated by *PTEN* disruption and DAXX deregulation through nuclear functions.

Additionally, our studies show that *DAXX* inhibition in GBM-PDX neurospheres suppresses tumour growth and increases survival, specifically in a *PTEN-*deficient background, in part by negatively regulating the expression of oncogenes implicated in gliomagenesis. It has been shown that SOX2, MYC and OLIG2 are required to maintain proliferation in progenitor cells and they have been implicated in different types of cancer^28^,^51^,^52^,^53^,^60^. We suggest that downregulation of expression of these GBM-TFs can be associated with the PTEN–DAXX–H3.3 complex, since H3.3 is enriched near the transcription binding sites of these genes, as we observed from the genomic data and in concordance with the literature^12^, and DAXX disruption only affects their expression in *PTEN-*deficient cells.

In a therapeutic context, DAXX–H3.3 interaction can be disrupted in *PTEN-null* cells using staple peptides as reported by Kim and collegues^61^ for the disassociation of an EZH2-EDD complex in a leukaemia model; or by using small molecules which have been efficient at antagonizing chromatin associated proteins and their interactions with other proteins^62^. Another strategy to target *DAXX* is by inhibiting its expression at the transcriptional level which can be attempted by performing high-throughput gene expression modulation by small molecules (GEMS) screening of compounds^28^,^63^.

In summary, we propose that PTEN–DAXX–H3.3 is a chromatin complex that regulates gene transcription (Fig. 6h and Supplementary Fig. 21). Our study nominates DAXX as a new therapeutic target to revert tumorigenesis caused by *PTEN* loss of function in GBMs. Additionally, a DAXX-inhibition strategy offers an opportunity for other *PTEN-null* tumours where MYC and CYCLIN-D1 are upregulated, including medulloblastomas, endometrial cancer, breast cancer and melanoma^8^,^46^,^47^.

## Methods

### Reagents and antibodies

A detailed description of the antibodies, drugs and kits used in this work is presented in Supplementary Methods.

### Cell culture and plasmids

MEFs and human glioma cell lines were cultured in DMEM plus 10% of fetal bovine serum (GIBCO/Life Technologies). Human glioblastoma patient-derived (GBM-PDX) spheres were maintained in DMEM/F12 1:1 medium with B27 supplement (GIBCO/Life Technologies) plus human recombinant EGF (20 ng ml^−1^) and FGF (10 ng ml^−1^) (Stemcell Technologies). A detailed description of MEFs, glioma cells, GBM-PDX spheres and vectors is in Supplementary Methods.

### *In vitro* pull-down assay

Fifty nanograms of human recombinant His-PTEN (ENZO Life Science) proteins were previously immobilized on 30 μl of nickel-beads (GE Healthcare Life Sciences) and then incubated with 200 ng of human recombinant Flag-DAXX protein (OriGene Technologies) for 2 h at 4 °C. Bound proteins were eluted and visualized by immunoblotting.

### Immuno-precipitation experiments

For immune-precipitation analysis, whole-cell lysates or nuclear fractions were extracted following the manufacturer’s instruction provided with the Universal Magnetic co-IP kit (54002, Active Motif). 300 μg of proteins were immunoprecipitated using 2.0 μg of antibody and 20 μl of Dynabeads (10007D, life technologies).

### RNA isolation and RT–QPCR

Total RNA was isolated with RNeasy kit (Qiagen), quantified and 1 μg of RNA was reverse-transcribed with the Superscript II reagent (Invitrogen). Q-PCR was performed using SYBER Green mix (Bio-Rad). Primers are described in Supplementary Methods.

### Lentivirus production and purification

Mission shRNA lentiviral particles (Sigma-Aldrich) were produced by co-transfection of shRNA pLKO-base lentivirus targeting *Daxx* or shRNA control vector, packaging gene vector pDELTA-8.9 and viral envelope vector pVSV-G in HEK293 cells with Lipofectamine 2000 (Life Technologies). A detailed description of protocol is in Supplementary Methods.

### Chromatin immunoprecipitation

Chromatin was isolated from 2 million cells according to the manufacturer’s recommended procedure in CHIP-IT kit (Active Motif 53040). Sheared chromatin (250 μg) was immunoprecipitated using 5 μg of ChIP quality antibody. ChIP-DNA was eluted (200 μl of elution buffer for ChIP-PCR or in 50 μl for ChIP-seq), and 2 μl were analysed by q-PCR using SYBER Green mix (Bio-Rad). Sheared chromatin (25 μg) were used as Input-DNA. Primers were designed by Prime3 and validated in the Genome Browser. Primers are described in Supplementary Methods.

### Cellular fractionation

Subcellular protein fractions were extracted according to the manufacturer’s instruction (Thermo Scientific) and 10 μg of proteins were resolved by SDS–PAGE followed by immunoblotting.

### Immunofluorescence microscopy

Cells were plated on poly-D-lysine-coated glass coverslips (Thermo Scientific), fixed with 10% formalin (Sigma-Aldrih), blocked with 2% of BSA IgG-free (Jackson ImmunoResearch) and stained with primary antibodies overnight at 4 °C. Secondary antibody was added for 1 h at room temperature. Coverslips were mounted on microscope glass slides using Fluro-Gel with DAPI (Electron Microscopy Science) followed by visualization using confocal microscopy (Leica SP5 confocal with resonant scanner). A detailed description of immunofluorescence acquisition and analysis is in Supplementary Methods.

### Cell proliferation and cell cycle analysis

One thousand cells were grown in 96-well plates and 72 h later cell proliferation was analysed by WST1 (MK400, Clontech) or ATplite assay (6016941, Perkin-Elmer). For cell cycle progression, cells were fixed with 70% of ethanol overnight and stained with FxCycle PI/RNase staining solution (F10797, Life Technologies). DNA content was evaluated by flow cytometry (FACS).

### Promoter reporter assay

A 1.5 and 1.0 Kb fragment upstream of the transcriptional start site of the human *CCND1* and *MYC* genes, respectively, were cloned into the pLightSwicth vector (32001, Active Motif). A detailed description of the assay is in Supplementary Methods.

### Sphere formation assay

Glioma stem cells were dissociated into single cells and 100 or 500 cells per well were plated in 96-well plates. Total number of spheres and total number of cells, per well and per treatment, were determined after 14 days in culture. A detailed description of the analysis is in Supplementary Methods.

### Flow cytometry analysis

Quantification of proteins associated to the fork replication complex was determined by flow cytometry. A detailed description of the method is in Supplementary Methods.

### Differentiation of GBM-PDX spheres

GBM spheres were dissociated into single cells and plated on glass coverslips coated with poly-D-lysine in DMEM medium with 1% of FBS. Coverslips were processed for immunostaining 7 days after plating.

### Lentivirus transduction of GBM-PDX spheres

Glioma spheres were co-transduced with purified lentivirus that encoded shRNAs anti-DAXX (shDAXX) or shRNA control (shLuc) at multiplicity of infection (MOI) 5, and a near infrared fluorescence protein (IRFP720, PerkinElmer) MOI 5, for 96 h.

### Intracranial xenograft tumour model

Animal research experiments were conducted under the regulations of the UCSD Animal Care Program, protocol number S00192M. GBM-PDX spheres were collected and resuspended at 0.5 or 1.0 × 10^6^ cells in 2 μl of PBS per animal, then stereotactically injected into the striatum (1.0 mm anteroposterior and 2.0 lateral from Bregma suture and 3 mm below the pial surface) of immunodeficient mice (Charles River laboratory).

### Tumour size measurement and survival analysis

Animals were observed for neurological signs and the relative fluorescence signal of the xenografts were analysed by fluorescence molecular tomography (PerkinElmer) and quantified using TrueQuant 3.1 software (PerkinElmer). For survival analysis, animals were killed when they showed signs of distress and morbidity.

### Densitometry quantification

Immunoblots were acquired with ChemiDocMP (Bio-Rad) and the intensity signal was quantified by densitometry analysis with Image Lab software.

### Immunohistochemistry and tissue microarray

Slides were deparaffinized and rehydrated by washing steps of 3 min in xylene, xylene:ethanol 1:1, 100% ethanol, 95% ethanol, 70% ethanol, 50% ethanol and water. After deparaffinization, sections were boiled in citrate buffer (pH 6.0) for 25 min. Sections were then treated with 5% serum-blocking solution for 20 min. A detailed description of the method is in Supplementary Methods.

### In silico protein–protein interactions

New PTEN nuclear interacting complexes were simulated using the bioinformatics site Human Interactome Map^31^. A detailed description of the analysis is in Supplementary Methods.

### Chromatin immunoprecipitation sequencing and analysis

Libraries were made with the Kapa Hyper Prep kit (Roche, KK8502), starting with 2.5 ng of IP DNA, ad amplified by 15 cycles of PCR amplification, according to the manufacturer’s protocol. Libraries were quantified and sized by running them on an Agilent Tapestation, measuring concentration of QPCR (Kapa Universal Library Quantification kit, Roche, KK4824). The libraries were run on an Illumina 2500, v4 chemistry, using a single read 50 protocol. ChIP-Seq analysis is described in Supplementary Methods.

### RNA sequencing and analysis

Total RNA was assessed for quality using an Agilent Tapestation, and all samples had RNA Integrity Numbers above 9.0. RNA libraries were generated using Illumina’s TruSeq Stranded mRNA Sample Prep Kit (Illumina, RS-122-2101) following the manufacturer’s instructions, modifying the shear time to 5 min. RNA-Seq analysis is described in Supplementary Methods.

### TCGA, REMBRANDT and Allen Brain analysis

Gene expression, genetic alterations and survival rate analysis from TCGA, REMBRANDT and Allen Human Brain Atlas are described in Supplementary Methods.

### Statistical analysis

Data sets were analysed by unpaired *t*-test or multiple comparisons one-way ANOVA or two-way ANOVA according to the experiment using GraphPad Prism software. *P<0.05, **P<0.001 and ***P<0.0001. Kaplan–Meier curves and comparison of survival were analysed using Long-rank (Mantel–Cox) test.

### Data availability

Data generated during the study have been deposited in Sequence Read Archive (SRA) SRP090820.

## Additional information

**How to cite this article:** Benitez, J. A *et al*. PTEN regulates glioblastoma oncogenesis through chromatin-associated complexes of DAXX and histone H3.3. *Nat. Commun.* **8**, 15223 doi: 10.1038/ncomms15223 (2017).

**Publisher’s note:** Springer Nature remains neutral with regard to jurisdictional claims in published maps and institutional affiliations.

## Supplementary Material

Supplementary Information Supplementary Figures, Supplementary Tables, Supplementary Methods and Supplementary References
