# Publisher Correction: PTEN regulates glioblastoma oncogenesis through chromatin-associated complexes of DAXX and histone H3.3

**DOI:** 10.1038/ncomms16217

**Published:** 2018-05-25

**Authors:** Jorge A. Benitez, Jianhui Ma, Matteo D’Antonio, Antonia Boyer, Maria Fernanda Camargo, Ciro Zanca, Stephen Kelly, Alireza Khodadadi-Jamayran, Nathan M. Jameson, Michael Andersen, Hrvoje Miletic, Shahram Saberi, Kelly A. Frazer, Webster K. Cavenee, Frank B. Furnari

Nature Communications 8:15223 doi: ; DOI: 10.1038/ncomms15223 (2017); Published 05 12 2017; Updated 05 25 2018

The originally published version of this Article contained an error in Fig. 1. In panel d, the uppermost western blot was inadvertently inverted during typesetting of the figure. This has now been corrected in both the PDF and HTML versions of the Article.

