# Supplementary material for: PTEN regulates glioblastoma oncogenesis through chromatin-associated complexes of DAXX and histone H3.3
Source: Nat Commun. 2017 May 12;8:15223. doi: 10.1038/ncomms15223 (PMC5437297; doi:10.1038/ncomms15223)
Supplement: Supplementary Information — Supplementary Figures, Supplementary Tables, Supplementary Methods and Supplementary References [file ncomms15223-s1.pdf]

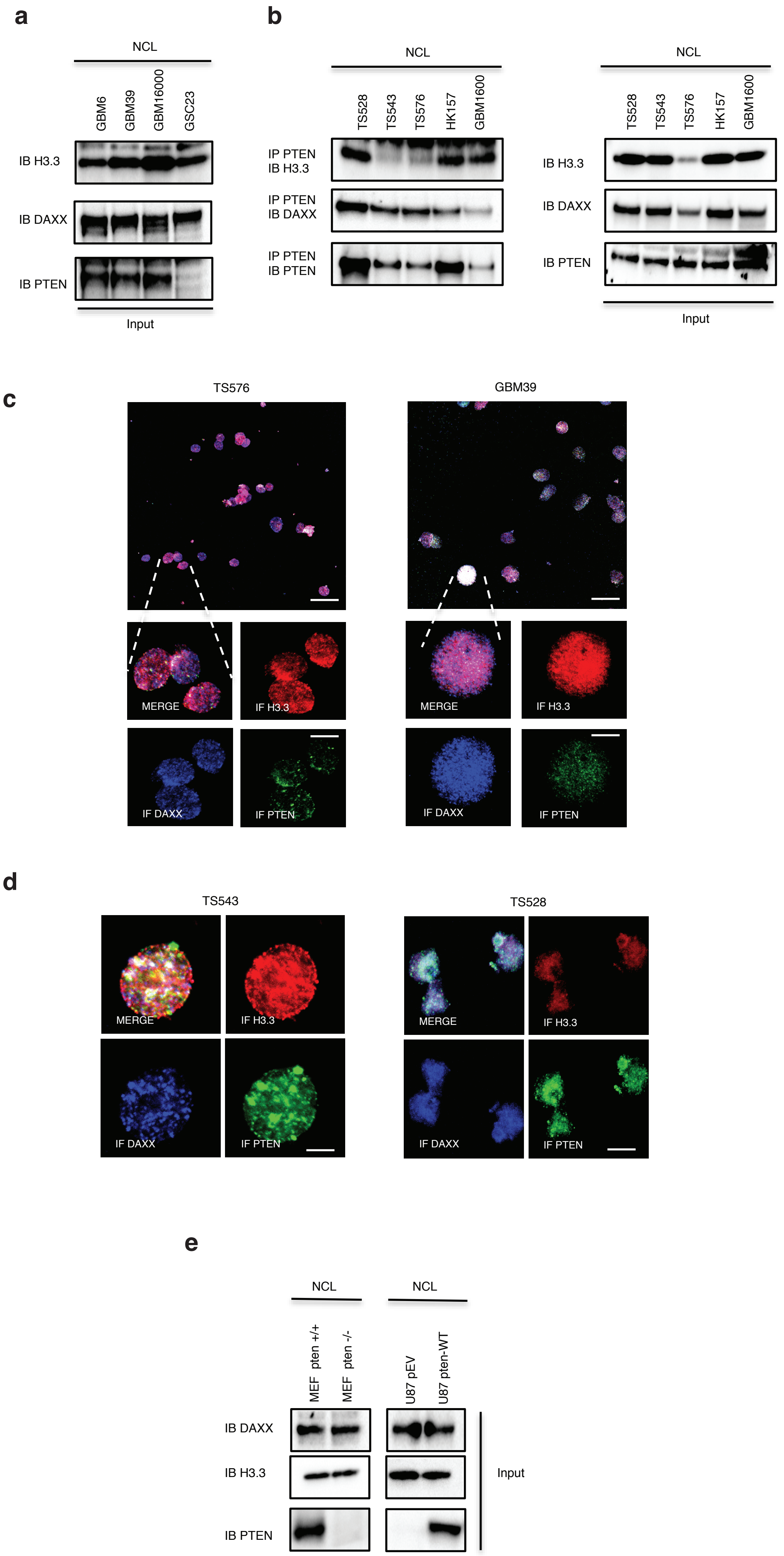
a
NCL
GBM6
GBM39
GBM16000
GSC23
IB H3.3
IB DAXX
IB PTEN
Input
b
NCL
TS528
TS543
TS576
HK157
GBM1600
IP PTEN IB H3.3
IP PTEN IB DAXX
IP PTEN IB PTEN
IB H3.3
IB DAXX
IB PTEN
Input
c
TS576
GBM39
MERGE
IF H3.3
IF DAXX
IF PTEN
d
TS543
TS528
MERGE
IF H3.3
IF DAXX
IF PTEN
e
NCL
MEF pten +/+
MEF pten -/-
U87 pEV
U87 pten-WT
IB DAXX
IB H3.3
IB PTEN
Input

Supplementary Figure 1. PTEN interacts with DAXX and H3.3. (a) Input samples from co-immunoprecipitation experiments of Figure 1a. (b) Co-immunoprecipitation analysis (left panel) and input samples (right panel) of PTEN, H3.3 and DAXX from GBM-spheres that express different levels of H3.3. (c) Confocal immunofluorescence analysis of endogenous PTEN, H3.3 and DAXX proteins in TS576 (left panel, Person's coefficient in colocalized region equal to 0.7055) and GBM39 (right panel, Person's coefficient in colocalized region equal to 0.4526) at two different magnifications. Scale bar in top panel, 50 uM. Scale bar in bottom panel, 5 uM. (d) Confocal immunofluorescence analysis of endogenous PTEN, H3.3 and DAXX proteins in TS543 (left panel, Person's coefficient in colocalized region equal to 0.7665) and TS528 (right panel, Person's coefficient in colocalized region equal to 0.7344). Scale bar, 5 uM. (e) Input samples from co-immunoprecipitation experiments of Figure 2d. NCL; nuclear protein extracts.

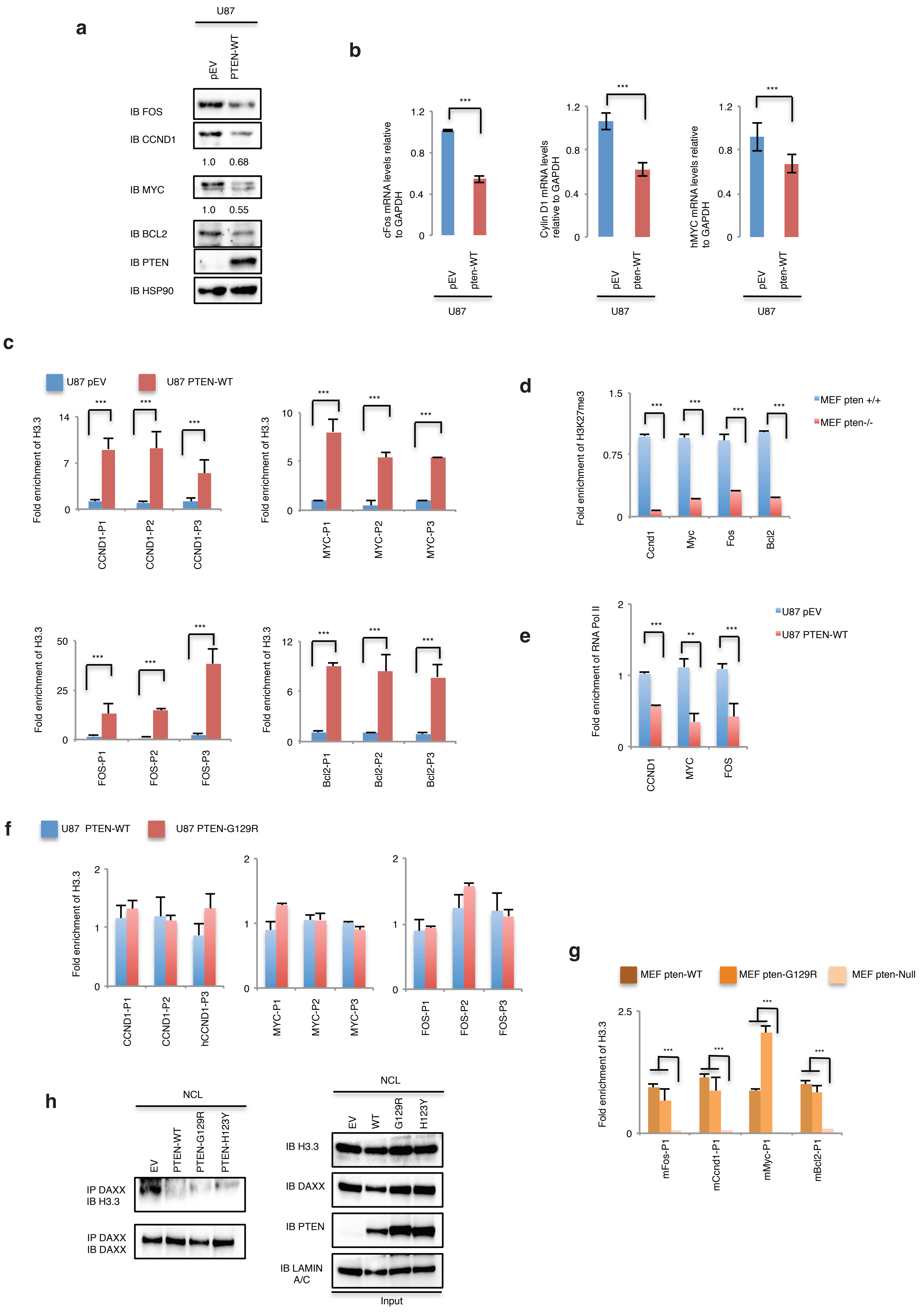

a
U87
pEV
PTEN-WT
IB FOS
IB CCND1
1.0 0.68
IB MYC
1.0 0.55
IB BCL2
IB PTEN
IB HSP90
b
cFos mRNA levels relative to GAPDH
Cylin D1 mRNA levels relative to GAPDH
hMYC mRNA levels relative to GAPDH
pEV
pten-WT
U87
c
U87 pEV
U87 PTEN-WT
Fold enrichment of H3.3
CCND1-P1
CCND1-P2
CCND1-P3
MYC-P1
MYC-P2
MYC-P3
FOS-P1
FOS-P2
FOS-P3
Bcl2-P1
Bcl2-P2
Bcl2-P3
d
Fold enrichment of H3K27me3
MEF pten +/+
MEF pten-/-
Ccnd1
Myc
Fos
Bcl2
e
Fold enrichment of RNA Pol II
U87 pEV
U87 PTEN-WT
CCND1
MYC
FOS
f
U87 PTEN-WT
U87 PTEN-G129R
Fold enrichment of H3.3
CCND1-P1
CCND1-P2
hCCND1-P3
MYC-P1
MYC-P2
MYC-P3
FOS-P1
FOS-P2
FOS-P3
g
MEF pten-WT
MEF pten-G129R
MEF pten-Null
Fold enrichment of H3.3
mFos-P1
mCcnd1-P1
mMyc-P1
mBcl2-P1
h
NCL
EV
PTEN-WT
PTEN-G129R
PTEN-H123Y
IP DAXX
IB H3.3
IP DAXX
IB DAXX
NCL
EV
WT
G129R
H123Y
IB H3.3
IB DAXX
IB PTEN
IB LAMIN A/C
Input

Supplementary Figure 2. PTEN represses oncogene expression by controlling H3.3 deposition on chromatin. (a and b) Gene expression analysis of FOS, CCND1, MYC and BCL2 by western blot (a) and RT-qPCR (b) in glioma cells stably transfected with empty vector (EV) or PTEN-WT. Error bars represent SEM from three different experiments. (c) Chromatin immuno-precipitation (ChIP) analysis with anti-histone H3.3 followed by qPCR in U87 glioma cells. (d) ChIP-qPCR with anti-H3K27me3 in pten+/+ and pten-/- MEF cells. (e) ChIP-qPCR with anti-RNA Polymerase II (RNA Pol II) in U87 cells stably transfected with empty vector (EV) or PTEN-WT. For ChIP assays, bar graphs indicate fold enrichment of H3K27me3 or RNA PolII over the input (n=3 biological samples with three replicates each, **p<0.001). (f and g) ChIP-qPCR analysis with anti-H3.3 in U87 (f) or MEF (g) cells expressing PTEN-WT or PTEN-G129R. For ChIP assays, bar graphs indicate fold enrichment of H3.3 over input (n=3 biological samples with three replicates each, *p<0.01, ***p<0.0001; NS: no significant differences). (h) Nuclear proteins (NCL) from U87 glioma cells transfected with empty vector, PTEN-WT or lipid phosphatase inactive PTEN-G129E and PTEN-H123Y mutants were immunoprecipitated with anti-DAXX followed by immunoblotting with anti-H3.3.

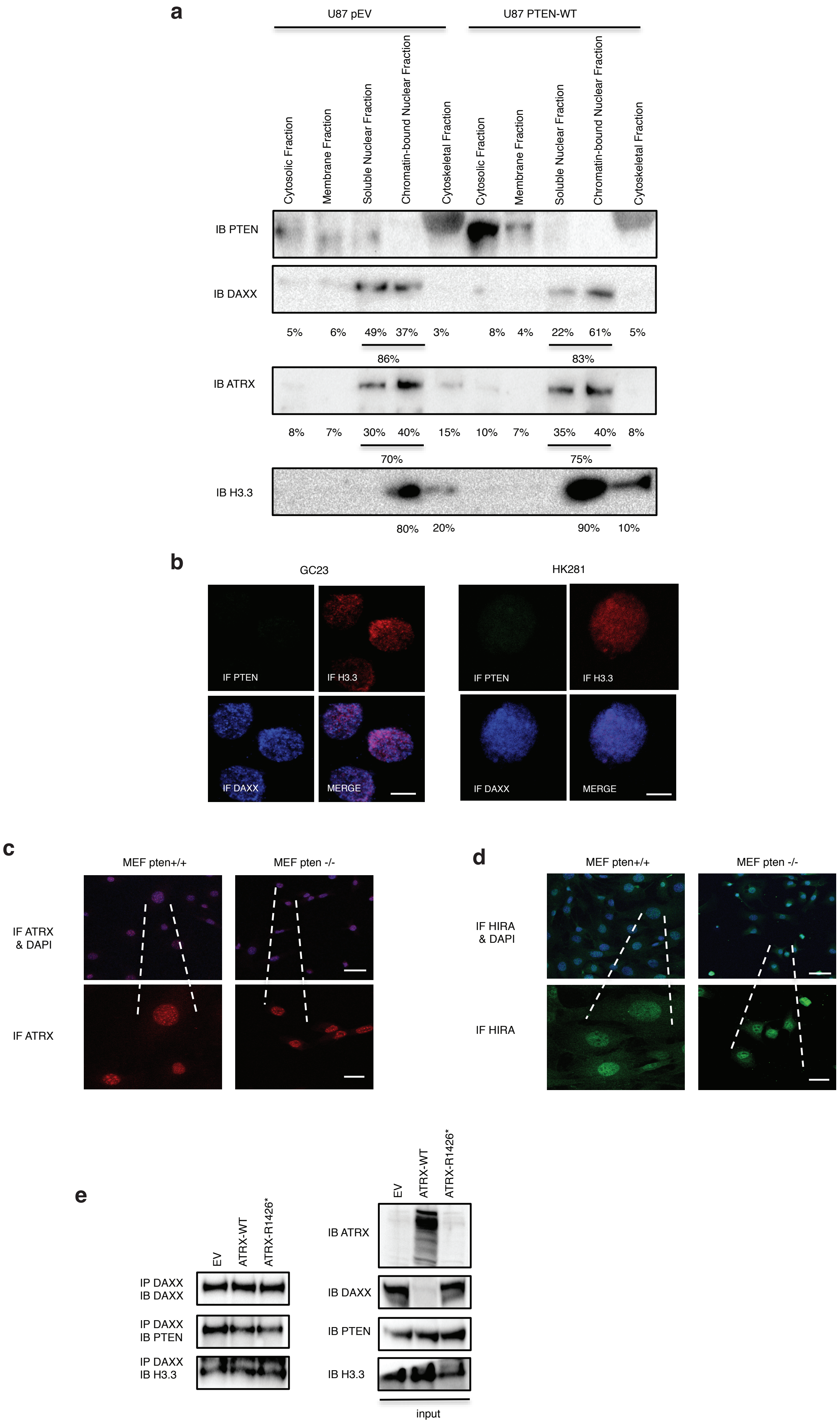
a
U87 pEV
U87 PTEN-WT
Cytosolic Fraction
Membrane Fraction
Soluble Nuclear Fraction
Chromatin-bound Nuclear Fraction
Cytoskeletal Fraction
IB PTEN
IB DAXX
5% 6% 49% 37% 3% 8% 4% 22% 61% 5%
86%
83%
IB ATRX
8% 7% 30% 40% 15% 10% 7% 35% 40% 8%
70%
75%
IB H3.3
80% 20% 90% 10%
b
GC23
HK281
IF PTEN
IF H3.3
IF DAXX
MERGE
c
MEF pten+/+
MEF pten -/-
IF ATRX & DAPI
IF ATRX
d
MEF pten+/+
MEF pten -/-
IF HIRA & DAPI
IF HIRA
e
EV
ATRX-WT
ATRX-R1426*
IP DAXX IB DAXX
IP DAXX IB PTEN
IP DAXX IB H3.3
IB ATRX
IB DAXX
IB PTEN
IB H3.3
input

Supplementary Figure 3. PTEN expression affects DAXX nuclear distribution. (a) Western blot of subcellular fractionations from U87-EV and U87 PTEN-WT. Numbers under the blots indicate percentage of protein signal in the different fractions determined by densitometry analysis. (b) Confocal immunofluorescence images of endogenous PTEN, H3.3 and DAXX in GSC23 (left panel) and HK281 (right panel) GBM-spheres. Scale bar, 5uM. (c and d) Representative immunofluorescence images of ATRX (c) or HIRA (d) signal co-stained with DAPI in MEF cells at two different magnifications. Top scale bar, 50 uM; bottom scale bar, 20 uM. (e) Immunoprecipitation (IP) assay from nuclear extracts of cells overexpressing ATRX-WT or ATRX-R1426* mutant. IP anti-DAXX followed by immunoblot (IB) anti-PTEN or anti-H3.3.

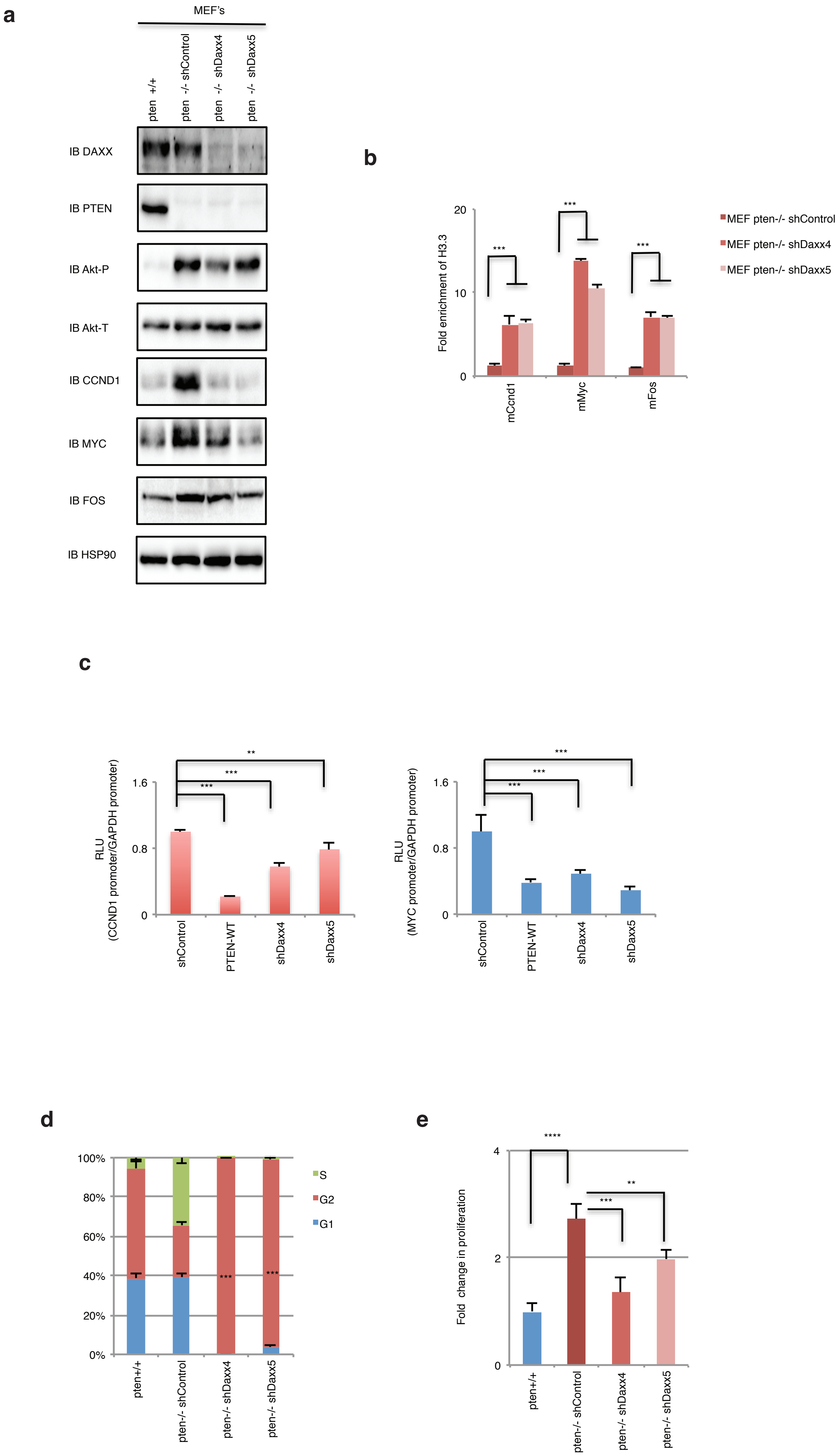
a
MEF's
pten +/+
pten -/- shControl
pten -/- shDaxx4
pten -/- shDaxx5
IB DAXX
IB PTEN
IB Akt-P
IB Akt-T
IB CCND1
IB MYC
IB FOS
IB HSP90
b
Fold enrichment of H3.3
MEF pten-/- shControl
MEF pten-/- shDaxx4
MEF pten-/- shDaxx5
mCcnd1
mMyc
mFos
c
RLU
(CCND1 promoter/GAPDH promoter)
RLU
(MYC promoter/GAPDH promoter)
shControl
PTEN-WT
shDaxx4
shDaxx5
d
S
G2
G1
pten+/+
pten-/- shControl
pten-/- shDaxx4
pten-/- shDaxx5
e
Fold change in proliferation

Supplementary Figure 4. DAXX knockdown compromises gene expression, cell cycle progression and cellular proliferation in PTEN-deficient cells. (a) Western blot analysis of total proteins from MEF pten -/- transduced with lentivirus anti-shRNA-Control or anti-shRNA-Daxx. HSP90 was used as a loading control. (b) ChIP-qPCR with anti-H3.3 from MEFs pten -/- expressing shRNAs targeting DAXX or shRNA control. (c) Promoter reporter assay of CCND1- (left panel) and MYC-promoter (right panel) in PTEN-deficient cells expressing shControl or shDAXX or over-expressing PTEN-WT. Relative luminescence signal from CCND1- or MYC-promoter was normalized to the GAPDH-promoter. RLU: relative luminescence units (n=3 biological samples with four replicates each, **p<0.001 and ***p<0.0001). (d) Cell cycle analysis of MEFs pten-/- with shControl or shDaxx knockdown. (e) Cell proliferation analysis in shControl or shDaxx MEF pten-/- cells. Error bars represent SEM from three different experiments (n=3 biological samples with three or six replicates each. **p<0.001 and ***p<0.0001).

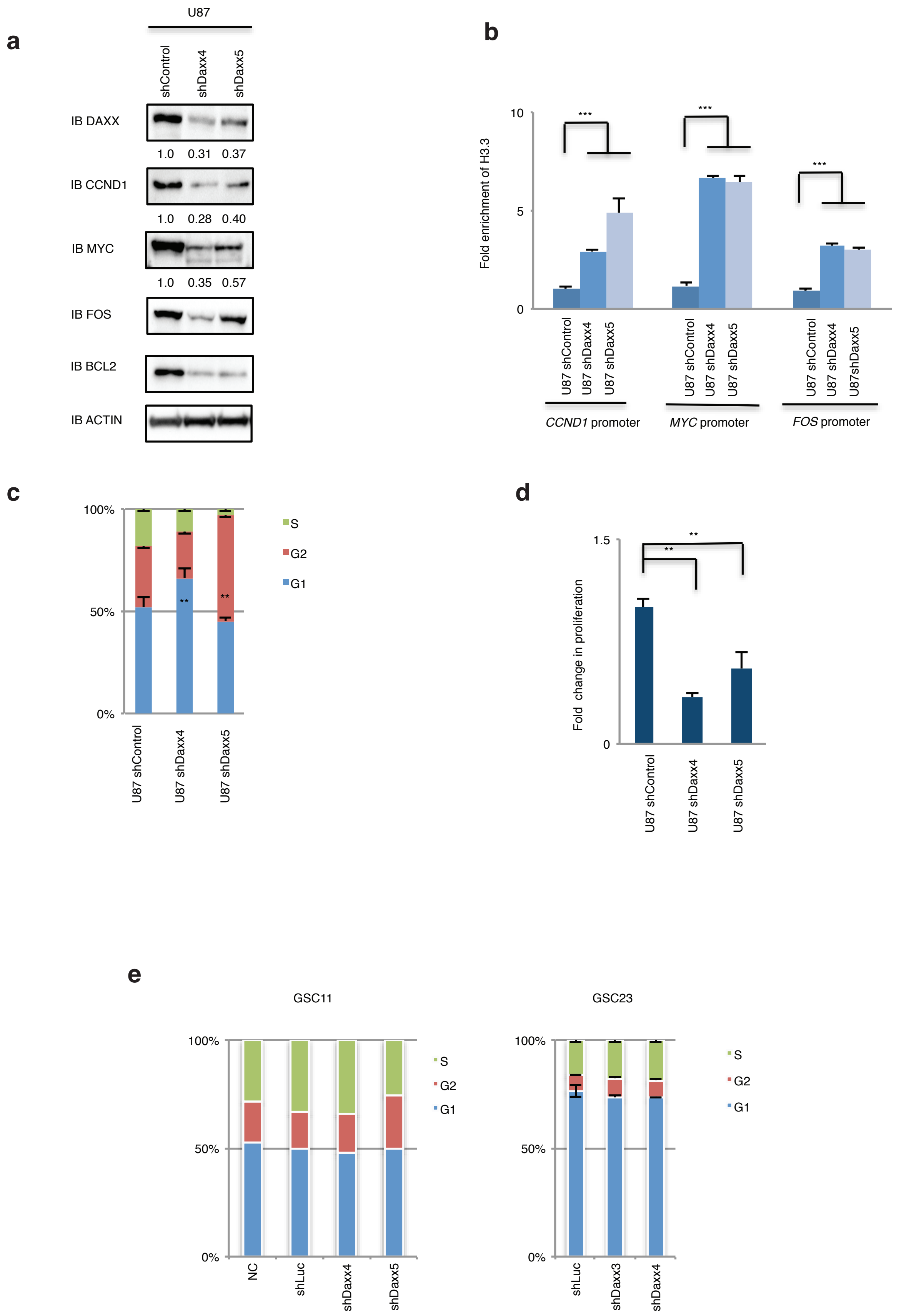
a
U87
shControl
shDaxx4
shDaxx5
IB DAXX
1.0 0.31 0.37
IB CCND1
1.0 0.28 0.40
IB MYC
1.0 0.35 0.57
IB FOS
IB BCL2
IB ACTIN
b
Fold enrichment of H3.3
***
U87 shControl
U87 shDaxx4
U87 shDaxx5
CCND1 promoter
MYC promoter
U87shDaxx5
FOS promoter
c
S
G2
G1
U87 shControl
U87 shDaxx4
U87 shDaxx5
d
Fold change in proliferation
**
e
GSC11
GSC23
NC
shLuc
shDaxx4
shDaxx5
shDaxx3

Supplementary Figure 5. DAXX inhibiton compromises gene expression and cellular proliferation in PTEN-deficient glioma cells. (a) Total proteins were analyzed by Western blot in PTEN-deficient glioma cells transduced with lentivirus shRNA control or two different shRNAs targeting DAXX. Actin is a loading control. Numbers under the blots indicate fold ratios of protein levels relative to shControl after normalization to Actin. (b) ChIP-qPCR anti-H3.3 was performed in PTEN-deficient glioma cells with stable knockdown of DAXX (shDaxx) or with shControl. For ChIP assays, bar graphs indicate fold enrichment of H3.3 over the input (n=3 biological samples with three replicates each, *** p<0.0001). (c) Cell cycle analysis of PTEN-deficient glioma cells after knockdown of DAXX (shDaxx) or shControl. (d) Cell proliferation analysis in PTEN-deficient glioma cells with shDaxx or shControl. (e) Cell cycle analysis in GSC11 (left panel) and GSC23 (right panel) GBM-spheres expressing shControl or shDaxx.

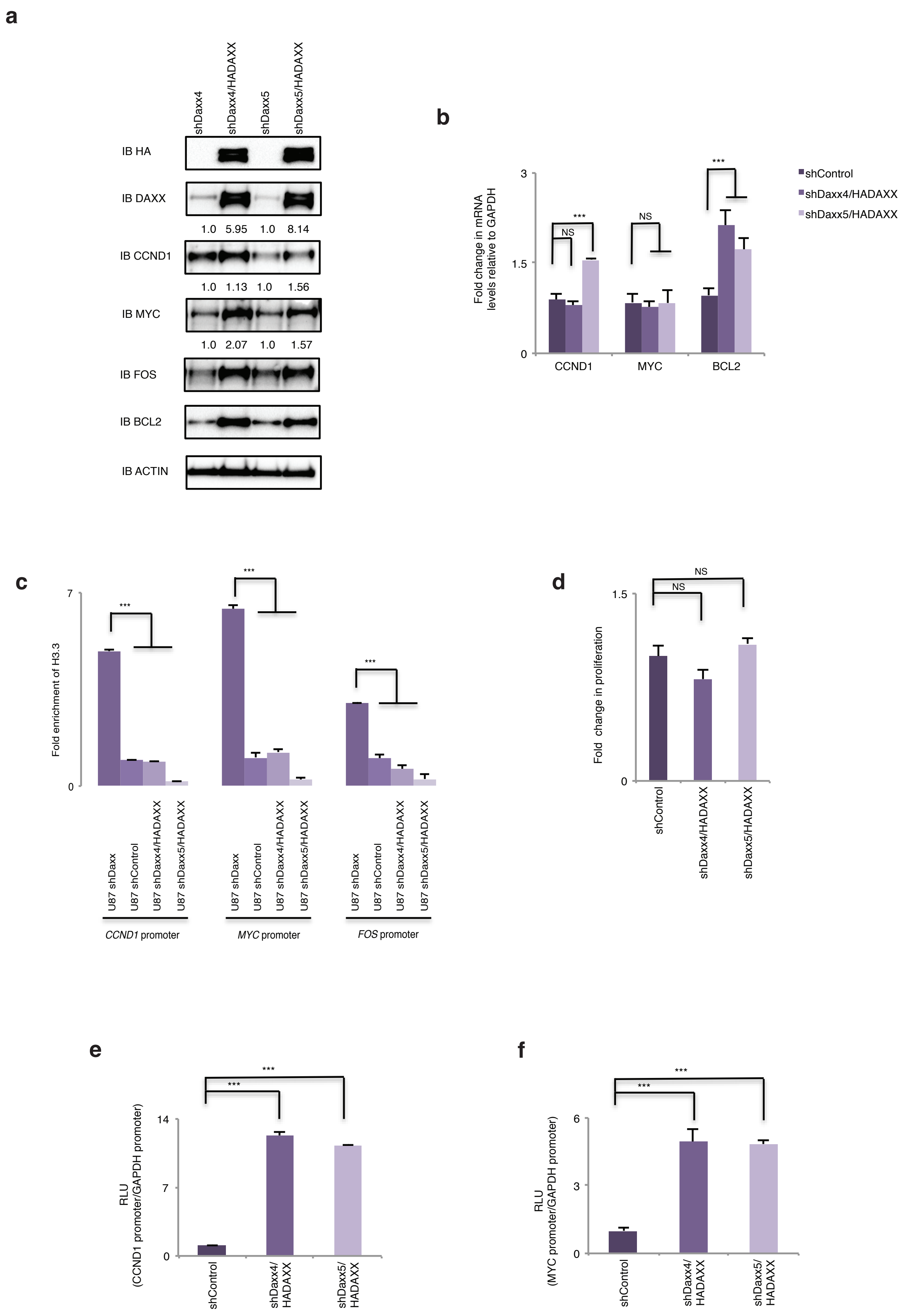

a
shDaxx4
shDaxx4/HADAXX
shDaxx5
shDaxx5/HADAXX
IB HA
IB DAXX
1.0 5.95 1.0 8.14
IB CCND1
1.0 1.13 1.0 1.56
IB MYC
1.0 2.07 1.0 1.57
IB FOS
IB BCL2
IB ACTIN
b
Fold change in mRNA levels relative to GAPDH
shControl
shDaxx4/HADAXX
shDaxx5/HADAXX
CCND1
MYC
BCL2
NS
***
c
Fold enrichment of H3.3
U87 shDaxx
U87 shControl
U87 shDaxx4/HADAXX
U87 shDaxx5/HADAXX
CCND1 promoter
MYC promoter
FOS promoter
d
Fold change in proliferation
shControl
shDaxx4/HADAXX
shDaxx5/HADAXX
e
RLU (CCND1 promoter/GAPDH promoter)
shControl
shDaxx4/ HADAXX
shDaxx5/ HADAXX
f
RLU (MYC promoter/GAPDH promoter)

Supplementary Figure 6. In vitro rescue experiments in glioma cells after re-expression of a DAXX shRNA-resistant (HA-DAXX) construct in Daxx knockdown cells. (a) Immunoblot analysis in PTEN-deficient cells expressing shDAXX before and after transfection with HA-DAXX-shRNA-resistant vector. Numbers under the blots indicate fold ratio of protein signal relative to control cells after normalization to the actin loading control. (b) mRNA expression analysis of CCND1, MYC and BCL2 by RT-qPCR in shControl or Daxx-kd cells after restoration of DAXX expression. Error bars represent SEM from three different experiments. (c) ChIP-qPCR of H3.3 in DAXX-kd cells expressing a HA-DAXX-shRNA-resistant vector. (d) Cell proliferation analysis in shControl and shDaxx cells expressing DAXX-shRNA-resistant. (e and f) Reporter assay using CCND1 (e) and MYC (f) promoters in shControl and shDAXX cells after re-expression of HA-DAXX vector. Relative luminescence signal from CCND1- or MYC-promoter was normalized to the GAPDH-promoter. RLU: relative luminescence units; NS: no significant differences. Error bars represent SEM from three different experiments (n=3 biological samples with three or six replicates each. ***p<0.0001).

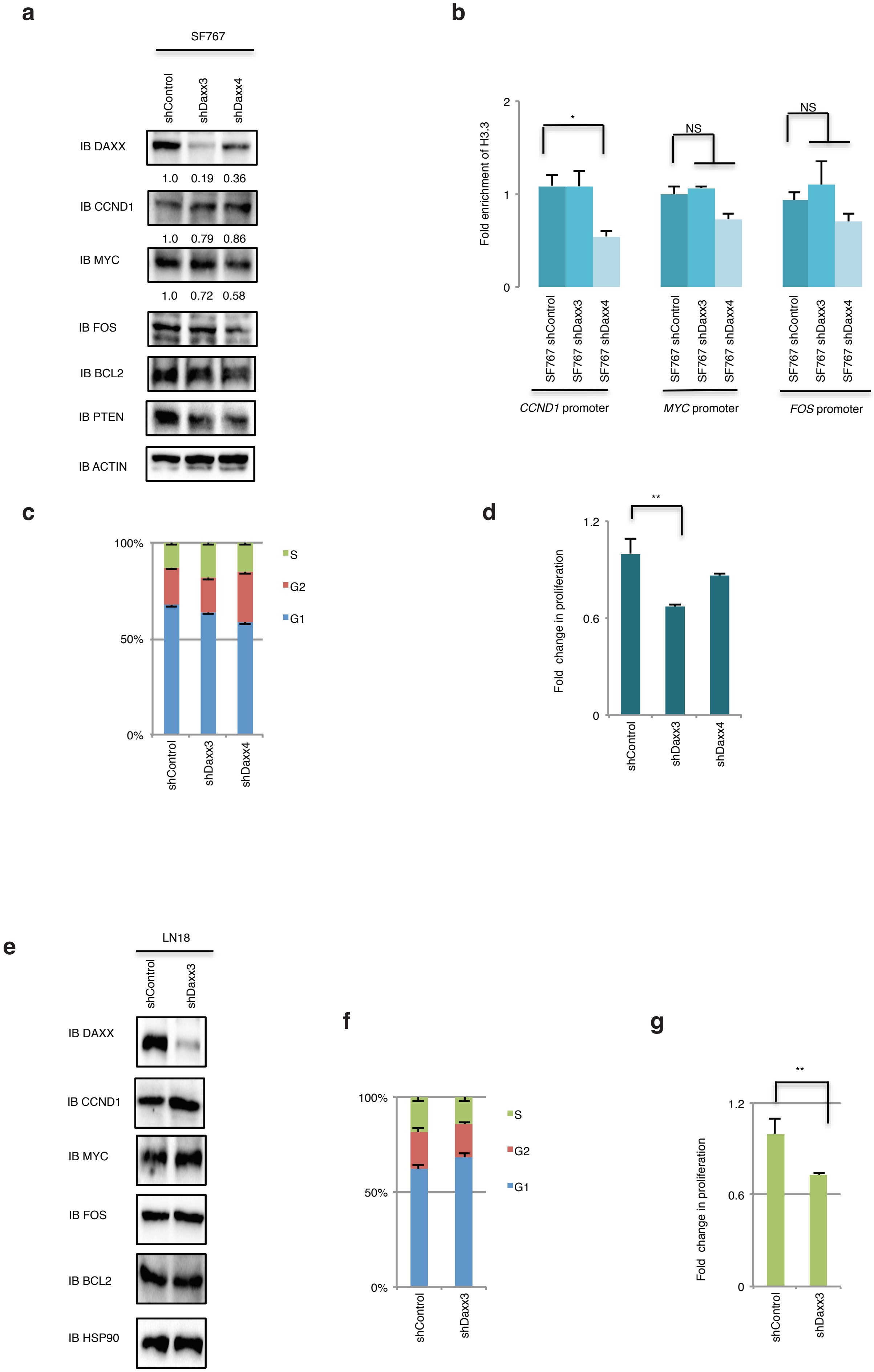
a
SF767
shControl
shDaxx3
shDaxx4
IB DAXX
1.0 0.19 0.36
IB CCND1
1.0 0.79 0.86
IB MYC
1.0 0.72 0.58
IB FOS
IB BCL2
IB PTEN
IB ACTIN
b
Fold enrichment of H3.3
2
1
0
*
NS
NS
SF767 shControl
SF767 shDaxx3
SF767 shDaxx4
CCND1 promoter
MYC promoter
FOS promoter
c
100%
50%
0%
S
G2
G1
shControl
shDaxx3
shDaxx4
d
**
1.2
0.6
0
Fold change in proliferation
shControl
shDaxx3
shDaxx4
e
LN18
shControl
shDaxx3
IB DAXX
IB CCND1
IB MYC
IB FOS
IB BCL2
IB HSP90
f
100%
50%
0%
S
G2
G1
shControl
shDaxx3
g
**
1.2
0.6
0
Fold change in proliferation
shControl
shDaxx3

Supplementary Figure 7. DAXX knockdown does not affect gene expression and cell cycle proliferation in PTEN-WT cells. (a and e) Western blot of total proteins from SF767 (a) and LN18 (e) cells transduced with lentivirus shRNA-Control or shRNA-Daxx. Actin or HSP90 were used as a loading control. (b) ChIP-qPCR anti-H3.3 was performed in PTEN-WT glioma cells with stable knockdown of DAXX (shDaxx) or with shControl. For ChIP assays, bar graphs indicate fold enrichment of H3.3 over the input (n=3 biological samples with three replicates each, *p<0.01; NS: no significant differences). (c and f) Cell cycle analysis of SF767 (c) and LN18 (f) cells with shControl or shDaxx knock-down. (d and g) Cell proliferation analysis in SF767 (d) and LN18 (g) cells transduced with shControl and shDaxx lentivirus. Error bars represent SEM from three different experiments (n=3 biological samples with three or six replicates each. **p<0.001).

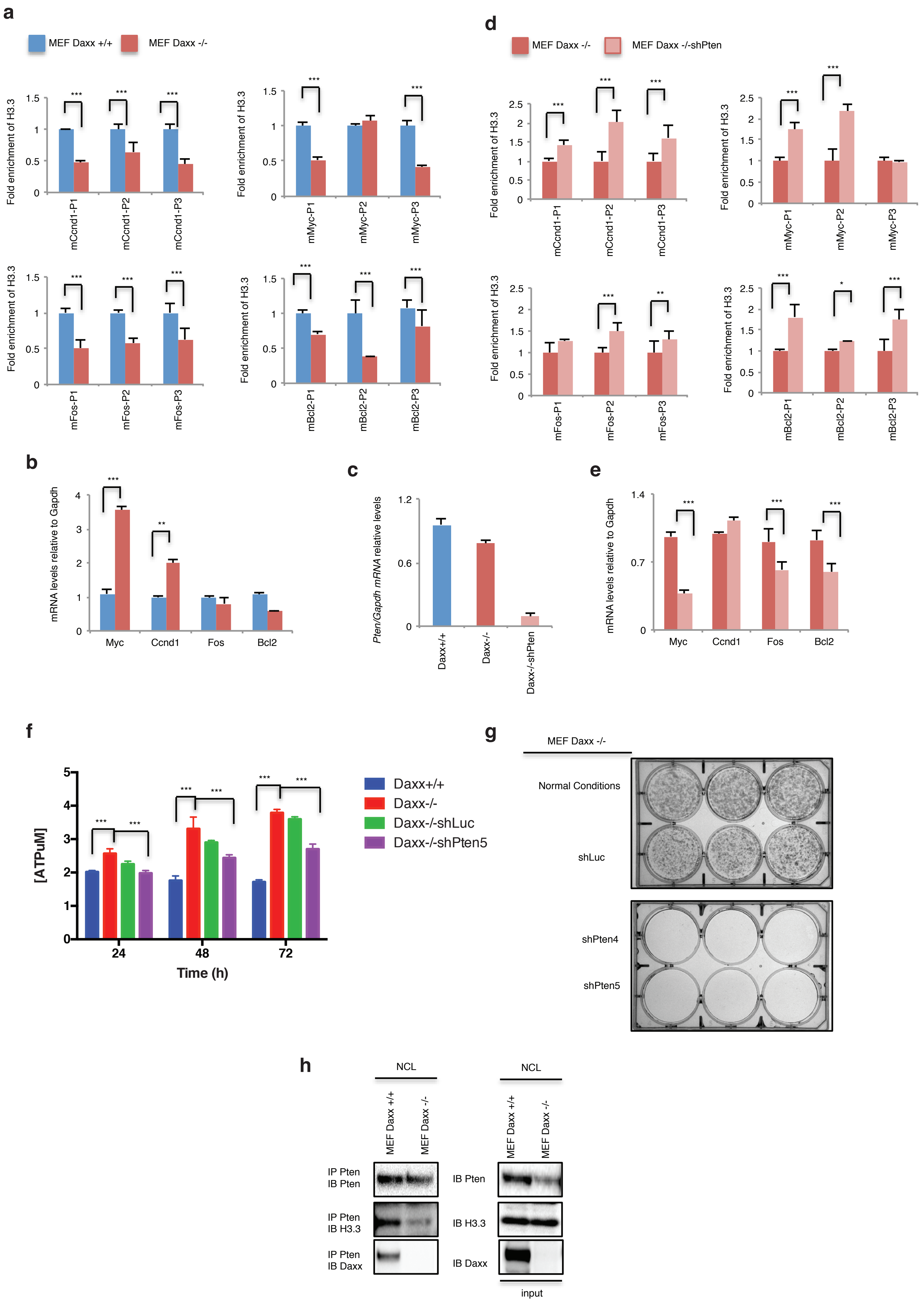
a
MEF Daxx +/+
MEF Daxx -/-
Fold enrichment of H3.3
mCcnd1-P1
mCcnd1-P2
mCcnd1-P3
mMyc-P1
mMyc-P2
mMyc-P3
mFos-P1
mFos-P2
mFos-P3
mBcl2-P1
mBcl2-P2
mBcl2-P3
d
MEF Daxx -/-
MEF Daxx -/-shPten
b
mRNA levels relative to Gapdh
Myc
Ccnd1
Fos
Bcl2
c
Pten/Gapdh mRNA relative levels
Daxx+/+
Daxx-/-
Daxx-/-shPten
e
mRNA levels relative to Gapdh
f
[ATPuM]
Time (h)
Daxx+/+
Daxx-/-
Daxx-/-shLuc
Daxx-/-shPten5
g
MEF Daxx -/-
Normal Conditions
shLuc
shPten4
shPten5
h
NCL
MEF Daxx +/+
MEF Daxx -/-
IP Pten
IB Pten
IP Pten
IB H3.3
IP Pten
IB Daxx
IB Pten
IB H3.3
IB Daxx
input

Supplementary Figure 8. Pten inhibition in Daxx-deficient MEFs restores H3.3 enrichment and affects oncogene expression. (a and d) ChIP-qPCR of H3.3 in Daxx-WT (Daxx +/+), Daxx–deficient (Daxx -/-) (a) and in Daxx-null/Pten-knockdown cells (Daxx -/-shPten) (d), using different sets of primers. For ChIP assays, bar graphs indicate fold enrichment of H3.3 over input (n=3 biological samples with three replicates each, ***p<0.0001). (b, c and e) mRNA expression analysis of Ccnd1, Myc, Fos, Bcl2 and Pten by RT-qPCR in Daxx-WT or Daxx–deficient cells (b and c) and after inhibition of Pten (e). (f) Cell proliferation analysis determined by ATP concentration at different time points. Error bars represent SEM from three different experiments (n=3 biological samples with three replicates each. **p<0.001 and ***p<0.0001). (g) Colony formation assay of Daxx-null, Daxx-/-shLuc and Daxx-/-shPten cells. (h) Nuclear proteins from MEF Daxx+/+ and MEF Daxx-/- were immunoprecipitated with anti-Pten followed by immunoblotting with anti-H3.3 or anti-Daxx.

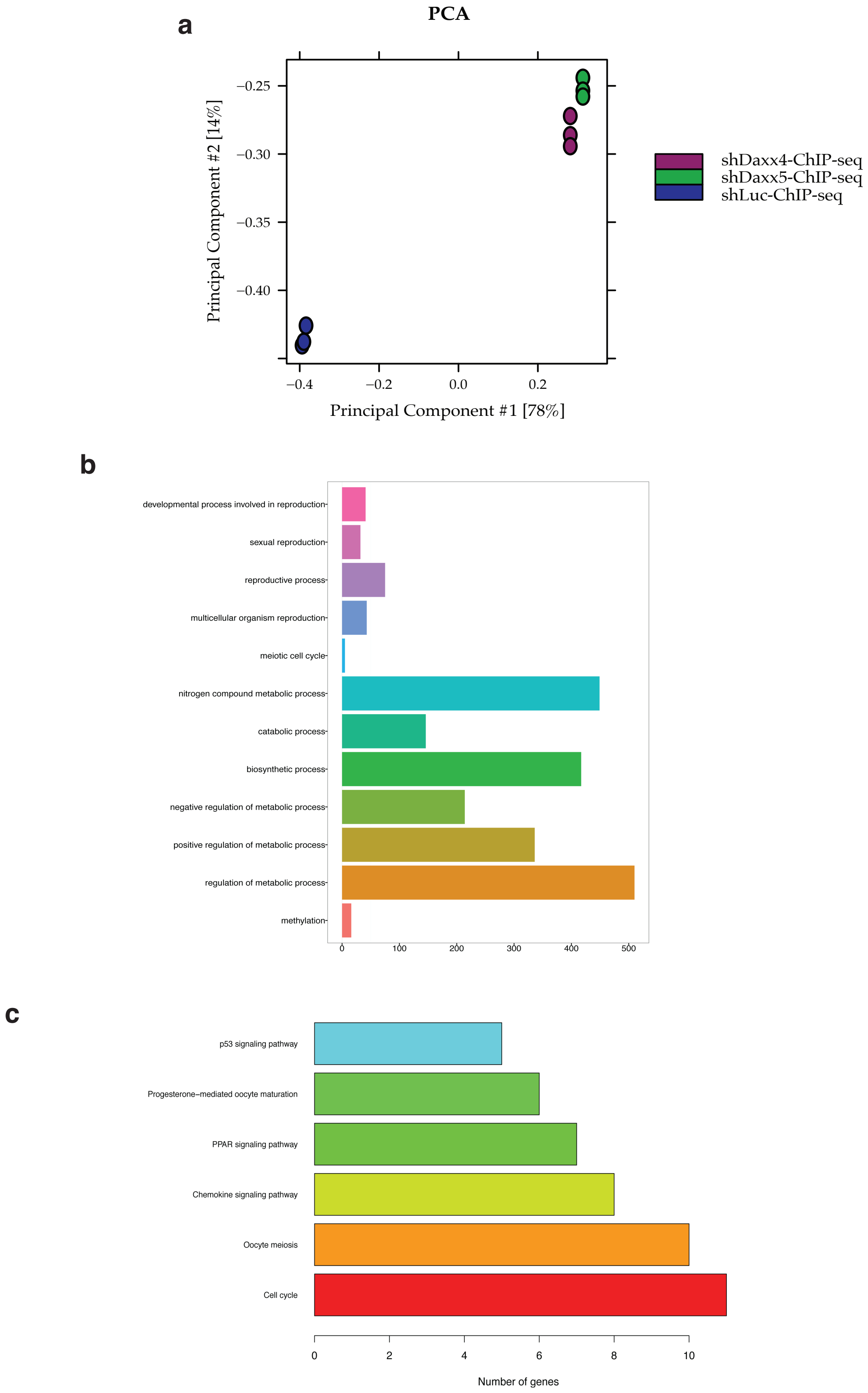

Supplementary Figure 9. H3.3 ChiP-seq analysis in DAXX-kd/PTEN-null GBM-PDX spheres. (a) Principal component analysis (PCA) of shControl (shLuc) and shDaxx (shDaxx4 and shDaxx5) PTEN-deficient GBM samples for ChIP-seq. (b and c) Gene ontology analysis of H3.3 differential binding genes in DAXX knockdown PTEN-deficient GBM-spheres, highlighting metabolic pathways (b), cell cycle and p53 signaling pathways (c).

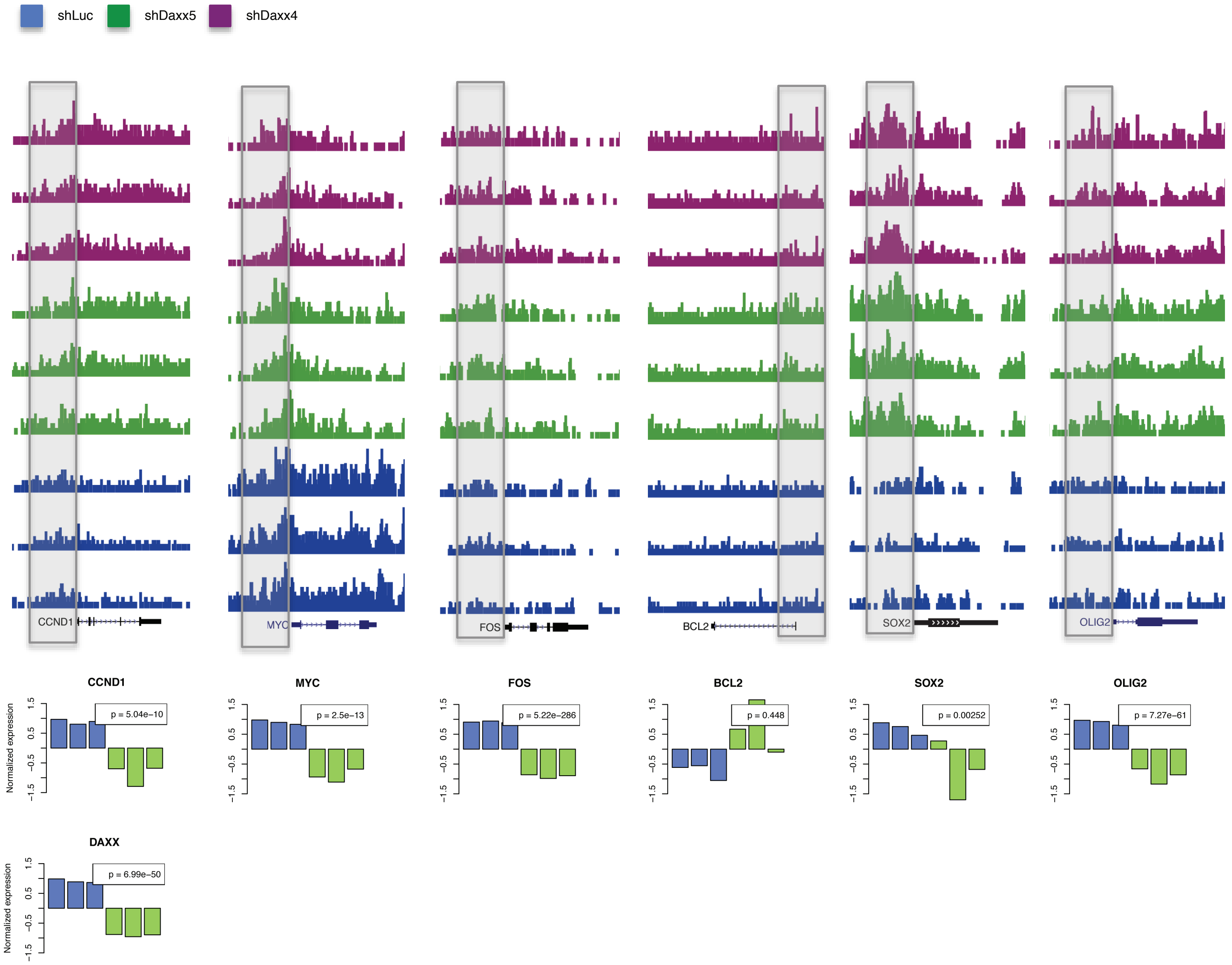

Supplementary Figure 10. DAXX inhibition affects H3.3 enrichment and oncogene expression. Top panel, H3.3 binding profile on indicated gene loci in shDaxx (shDaxx4 and shDaxx5) and shControl (shLuc) PTEN-deficient GBM-spheres. Bottom panel, Normalized gene expression of candidate genes in shLuc and shDaxx cells.

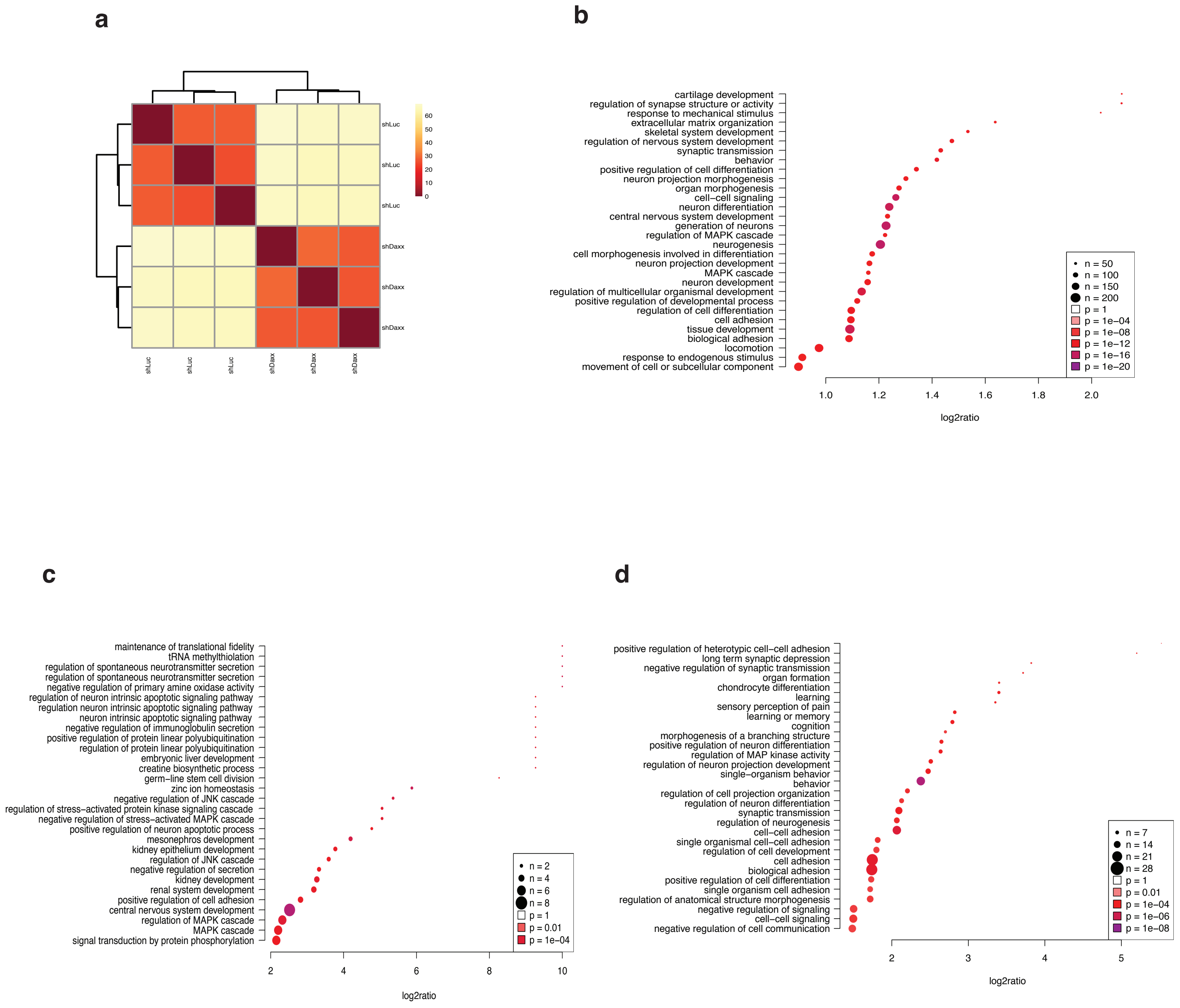

Supplementary Figure 11. Transcriptome analysis in DAXX-kd/PTEN-null GBM-PDX spheres. (a) Sample-to-sample distance matrix of shControl (shLuc) and shDaxx PTEN-deficient GBM samples for RNA-seq. (b to d) Gene ontology analysis of differentially expressed (DiffExp) genes between shLuc and shDaxx samples (b), upregulated genes with H3.3 enrichment (c) and downregulated genes with H3.3 enrichment (d).

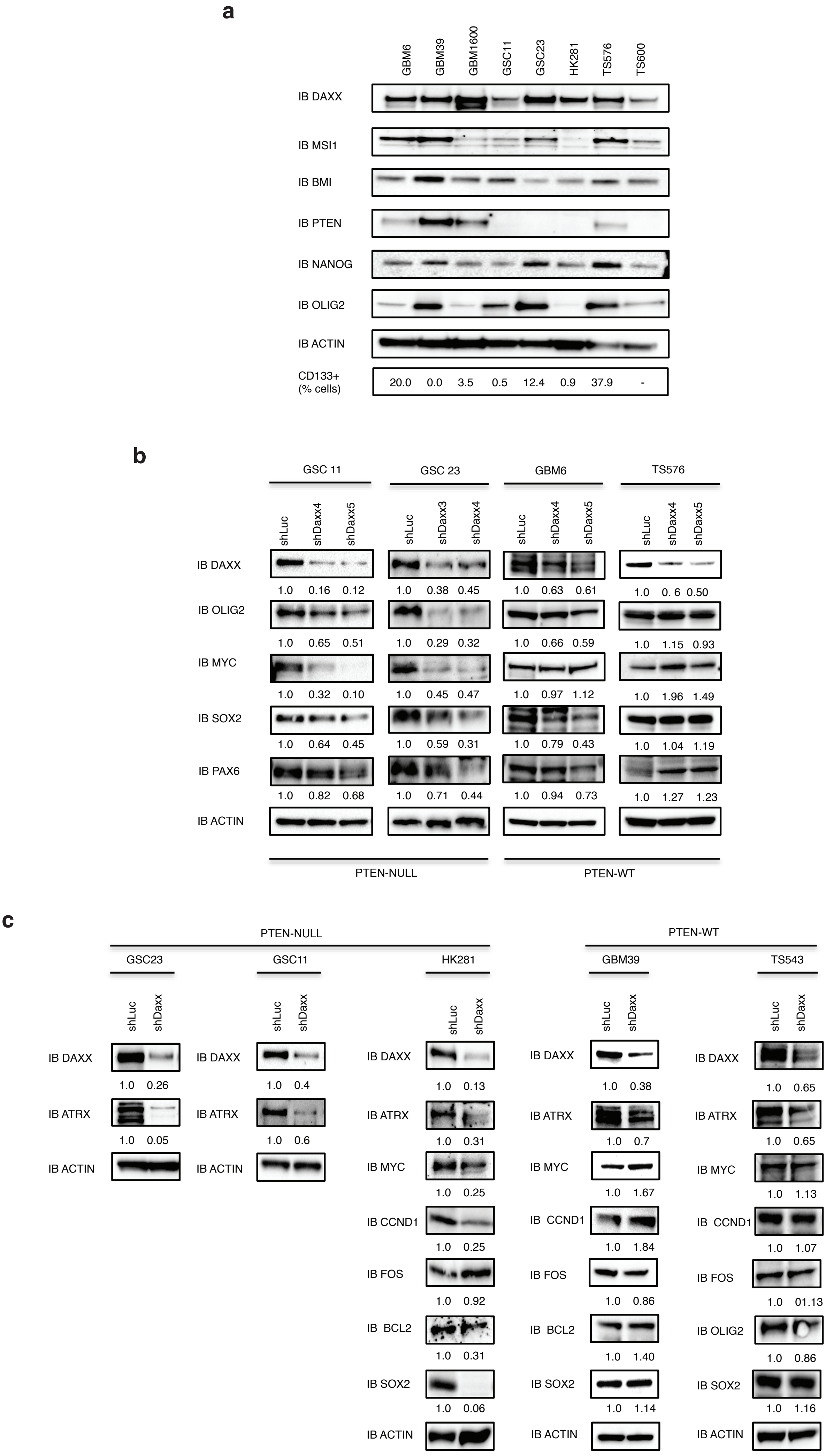
a
GBM6
GBM39
GBM1600
GSC11
GSC23
HK281
TS576
TS600
IB DAXX
IB MSI1
IB BMI
IB PTEN
IB NANOG
IB OLIG2
IB ACTIN
CD133+ (% cells)
20.0 0.0 3.5 0.5 12.4 0.9 37.9 -
b
GSC 11
GSC 23
GBM6
TS576
shLuc shDaxx4 shDaxx5
shLuc shDaxx3 shDaxx4
shLuc shDaxx4 shDaxx5
shLuc shDaxx4 shDaxx5
IB DAXX
1.0 0.16 0.12
1.0 0.38 0.45
1.0 0.63 0.61
1.0 0. 6 0.50
IB OLIG2
1.0 0.65 0.51
1.0 0.29 0.32
1.0 0.66 0.59
1.0 1.15 0.93
IB MYC
1.0 0.32 0.10
1.0 0.45 0.47
1.0 0.97 1.12
1.0 1.96 1.49
IB SOX2
1.0 0.64 0.45
1.0 0.59 0.31
1.0 0.79 0.43
1.0 1.04 1.19
IB PAX6
1.0 0.82 0.68
1.0 0.71 0.44
1.0 0.94 0.73
1.0 1.27 1.23
IB ACTIN
PTEN-NULL
PTEN-WT
c
PTEN-NULL
PTEN-WT
GSC23
GSC11
HK281
GBM39
TS543
shLuc shDaxx
GSC23: IB DAXX 1.0 0.26; IB ATRX 1.0 0.05; IB ACTIN
GSC11: IB DAXX 1.0 0.4; IB ATRX 1.0 0.6; IB ACTIN
HK281: IB DAXX 1.0 0.13; IB ATRX 1.0 0.31; IB MYC 1.0 0.25; IB CCND1 1.0 0.25; IB FOS 1.0 0.92; IB BCL2 1.0 0.31; IB SOX2 1.0 0.06; IB ACTIN
GBM39: IB DAXX 1.0 0.38; IB ATRX 1.0 0.7; IB MYC 1.0 1.67; IB CCND1 1.0 1.84; IB FOS 1.0 0.86; IB BCL2 1.0 1.40; IB SOX2 1.0 1.14; IB ACTIN
TS543: IB DAXX 1.0 0.65; IB ATRX 1.0 0.65; IB MYC 1.0 1.13; IB CCND1 1.0 1.07; IB FOS 1.0 01.13; IB OLIG2 1.0 0.86; IB SOX2 1.0 1.16; IB ACTIN

Supplementary Figure 12. DAXX inhibition affects oncogenesis in PTEN-deficient patient-derived glioma neurospheres. (a) Western blot analysis in GBM-PDX spheres showing expression levels of proteins associated with oncogenesis. Also shown is the percentage of CD133 positive cells determined by flow cytometry analysis. (b) Protein analysis of patient-derived glioma (GBM-PDX) spheres that are PTEN null (GSC11 and GSC23) and PTEN wild type (GBM6 and TS576) stably transduced with shRNA lentivirus targeting DAXX (shDaxx) or shRNA control (shLuc). Protein expression was quantified by densitometry analysis. (c) Protein analysis of patient-derived glioma (GBM-PDX) spheres PTEN null (GSC23, GSC11 and HK281) and PTEN wild type (GBM39 and TS543) stably transduced with shRNA lentivirus targeting DAXX or shRNA control (shLuc). Protein expression was determined by immnunoblot and quantified by densitometry analysis. Numbers under the blots indicate fold ratios of protein levels relative to shControl after normalization to actin.

Confidence intervals for
1/(cell frequency)

Group	GSC 11	GSC 23	HK281	GBM6	TS576	
shRNA	PTEN-NULL	PTEN-NULL	PTEN-NULL	PTEN-WT	PTEN-WT	
shDaxx4	43.3	108.0	102	7.33	51.6	
shDaxx5	41.5	149.5	-	16.34	17.0	
shLuc	15.5	15.7	19	5.03	44.6	

Supplementary Figure 13. Table showing the frequency of GBM-PDX PTEN-WT and PTEN-deficient cells that form spheres by in vitro limiting dilution assay (confidence interval 0.95).

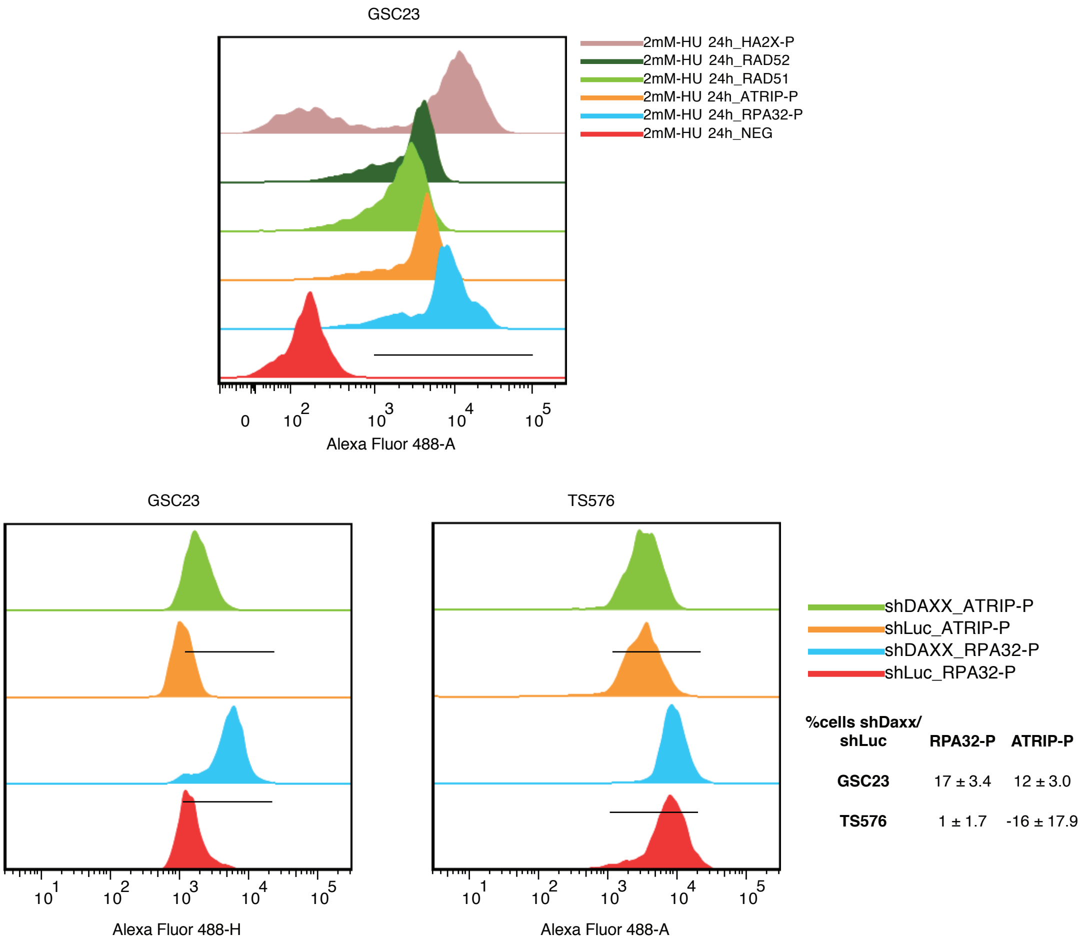

%cells shDaxx/ shLuc	RPA32-P	ATRIP-P
GSC23	17 ± 3.4	12 ± 3.0
TS576	1 ± 1.7	-16 ± 17.9

Supplementary Figure 14. DAXX inhibition affects DNA replication markers in PTEN-null GBM-spheres. Flow cytometry quantification of proteins associated to the fork replication complex. Top panel, histogram-plots showing positive populations corresponding to different markers of the fork replication complex (H2AX-P, RAD52, RAD51, ATRIP-P and RPA32-P) after treatment with 2mM of Hydorxyurea (HU). Bottom panel, representative histogram-plots and quantification of shDAXX positive populations to ATRIP-P and RPA32-P markers.

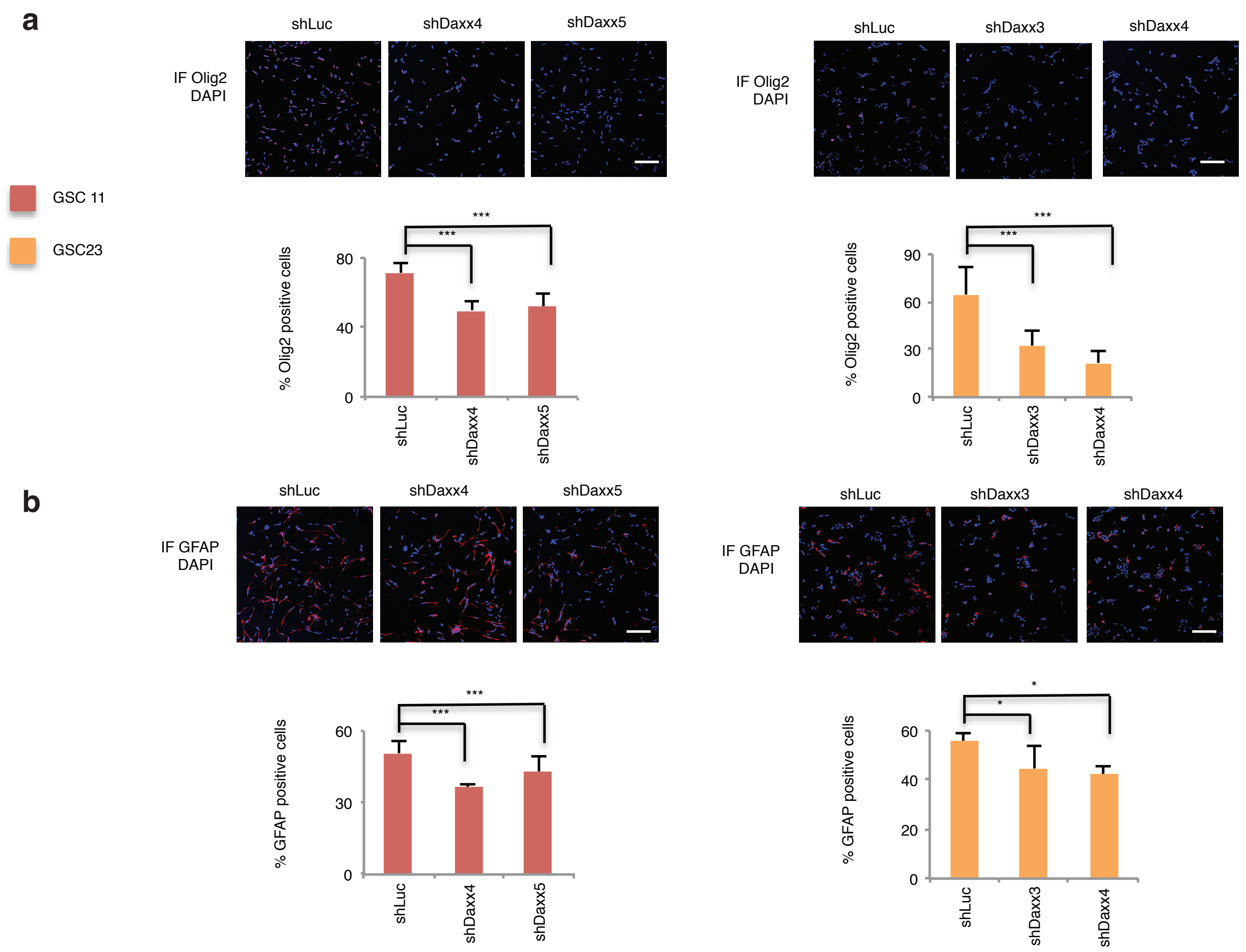

Supplementary Figure 15. DAXX inhibition affects differentiation capability of PTEN-null GBM-spheres. Immunofluorescence analysis of OLIG2 (a) and GFAP (b) positive cells in GBM-PDXs under differentiation conditions. Top panel, representative immunofluorescence images. Scale bar, 150 uM. Bottom panel, quantification of the percentage of cells positive for OLIG2 (a) or GFAP (b) (n=3 biological samples with six or twelve replicates each. Error bars represent SEM from three different experiments. (*$p<0.05$, **$p<0.001$ and ***$p<0.0001$).

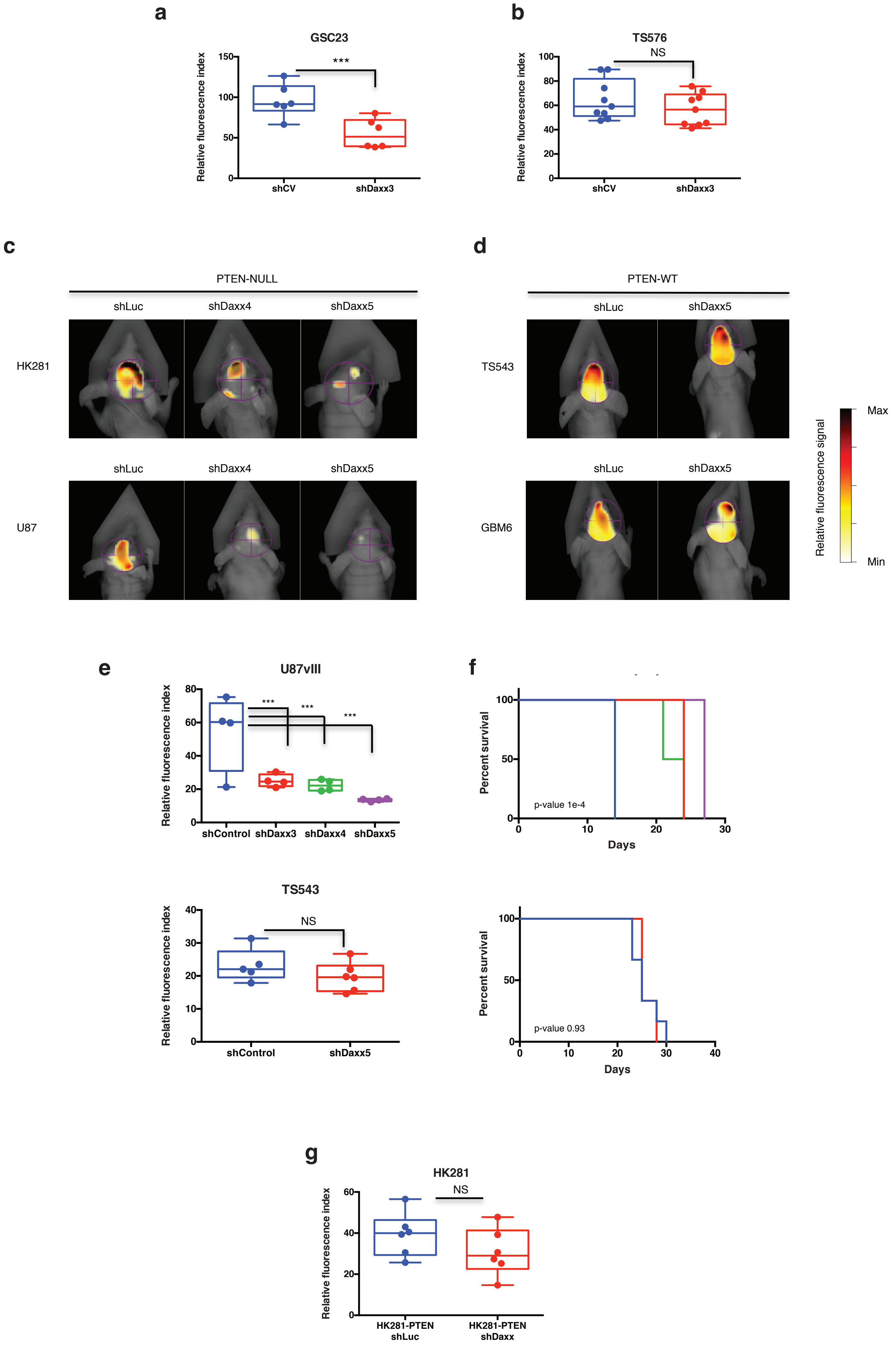
a
GSC23
***
Relative fluorescence index
150
100
50
0
shCV
shDaxx3
b
TS576
NS
Relative fluorescence index
100
80
60
40
20
0
shCV
shDaxx3
c
PTEN-NULL
shLuc
shDaxx4
shDaxx5
HK281
shLuc
shDaxx4
shDaxx5
U87
d
PTEN-WT
shLuc
shDaxx5
TS543
shLuc
shDaxx5
GBM6
Relative fluorescence signal
Max
Min
e
U87vIII
***
***
***
Relative fluorescence index
80
60
40
20
0
shControl
shDaxx3
shDaxx4
shDaxx5
f
Percent survival
100
50
0
p-value 1e-4
0
10
20
30
Days
TS543
NS
Relative fluorescence index
40
30
20
10
0
shControl
shDaxx5
Percent survival
100
50
0
p-value 0.93
0
10
20
30
40
Days
g
HK281
NS
Relative fluorescence index
60
40
20
0
HK281-PTEN shLuc
HK281-PTEN shDaxx

Supplementary Figure 16. DAXX knockdown inhibits tumor growth and increases survival rate in GBM PTEN-deficient models. (a and b) Relative fluorescence quantification of GSC23 PTEN-NULL (a) or TS756 PTEN-WT (b) engrafted mice with shControl or shDAXX. (c and d) FMT images of mice intracranially injected with GBM-PDX spheres (HK281, TS543 and GBM6) or glioma cells (U87) expressing shControl or shDAXX. (e) Relative fluorescence quantification of PTEN-NULL (top) or PTEN-WT (bottom) xenografts by FMT imaging in shControl or shDAXX. (f) Kaplan-Meier survival curves of mice implanted with PTEN-deficient (top) or PTEN-WT (bottom) cells expressing shDaxx or shControl. (g) Relative fluorescence quantification of HK281-PTEN engrafted mice after inhibition of DAXX (HK281-PTEN/shDaxx) or shControl (HK281-PTEN/shLuc) by FMT imaging. (***$p<0.0001$; NS: no significant differences).

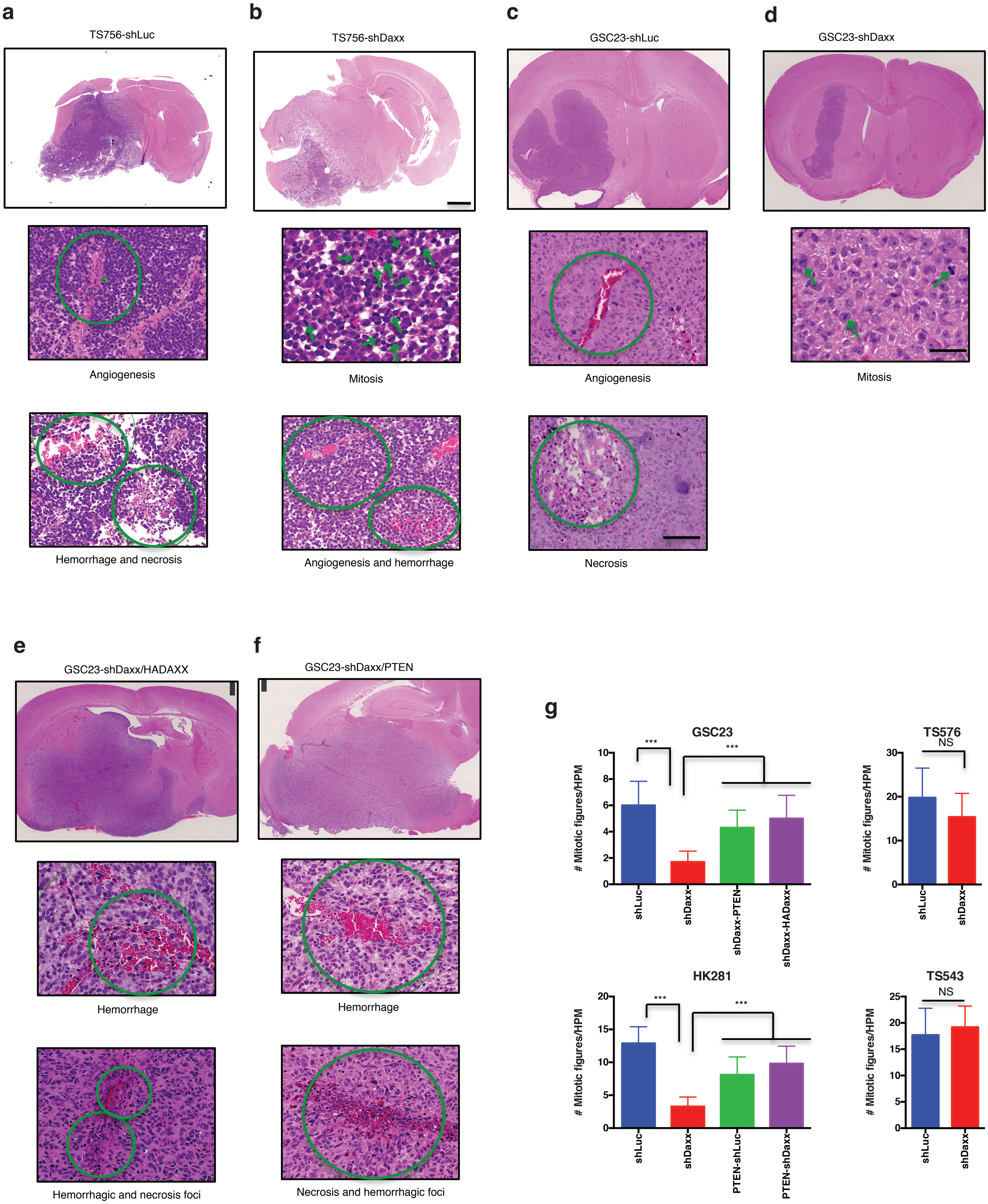
a
TS756-shLuc
Angiogenesis
Hemorrhage and necrosis
b
TS756-shDaxx
Mitosis
Angiogenesis and hemorrhage
c
GSC23-shLuc
Angiogenesis
Necrosis
d
GSC23-shDaxx
Mitosis
e
GSC23-shDaxx/HADAXX
Hemorrhage
Hemorrhagic and necrosis foci
f
GSC23-shDaxx/PTEN
Hemorrhage
Necrosis and hemorrhagic foci
g
GSC23
Mitotic figures/HPM
***
***
shLuc
shDaxx
shDaxx-PTEN
shDaxx-HADaxx
TS576
NS
shLuc
shDaxx
HK281
***
***
shLuc
shDaxx
PTEN-shLuc
PTEN-shDaxx
TS543
NS
shLuc
shDaxx

Supplementary Figure 17. Histological analyses of brain tumors from animals orthotopically engrafted with PTEN-WT (TS576) or PTEN-deficient (GSC23) GBM-spheres expressing shControl or shDaxx. Top panel, representative images of mouse brain cross sections (H&E, hematoxylin and eosin stained) showing the effect of shDaxx inhibition on brain tumors derived from PTEN-WT (TS576, a and b), PTEN-null (GSC23, c and d) and after re-expression of DAXX (e) or PTEN (f) in shDaxx/PTEN-null GBM-spheres (scale bar, 2.5mm). Middle and bottom panel, histological analysis of brain tumors derived (H&E stained) from TS576 (a and b), GSC23 (c and d), and GSC23/shDAXX reconstituted with DAXX (e) or PTEN (f). Green circles denote angiogenesis or necrosis or hemorrhagic areas in the tumor (scale bar, 100 uM). Green arrows indicate mitotic cells (scale bar, 50 uM). (g) Mitotic figures quantification at high power magnification (HPM, 40X) of different brain tumor xenograft conditions. Error bars represent SEM of 10 different fields from two cross sections mice brain per xenograft condition (***$p<0.0001$, NS: no significant differences).

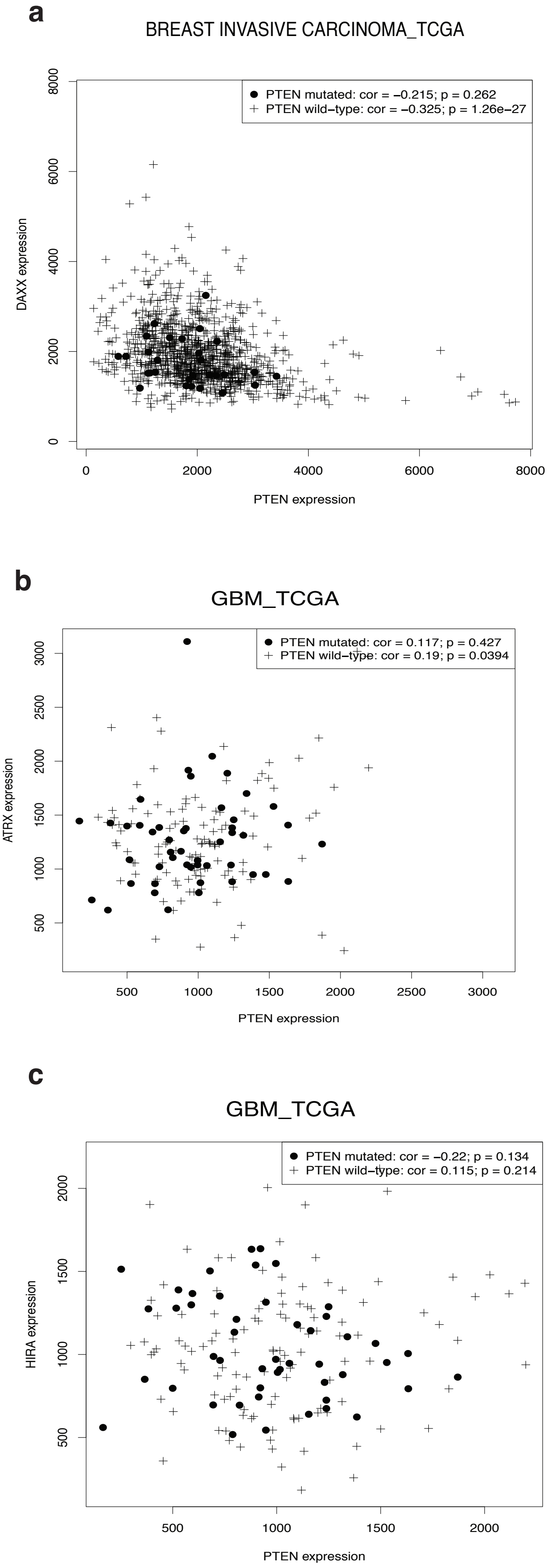

Supplementary Figure 18. Gene expression analysis from TCGA database. (a) Inverse correlated expression between DAXX and PTEN in invasive breast carcinoma from TCGA. (b and c) Gene correlation analysis between PTEN and ATRX (b) and PTEN and HIRA (c) in GBM-TCGA database. .

a

TCGA GBM
DAXX altered in 2 (1%) of 281 patients/cases

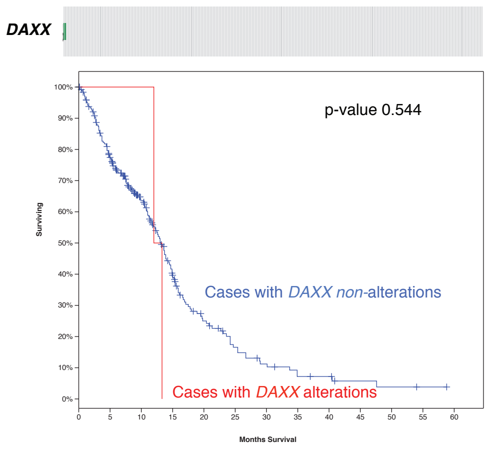

	Total cases	Cases deceased	Median months survival
**Cases with alterations**	2	2	12
**Cases without alterations**	244	161	13.1

b

TCGA GBM
DAXX and *PTEN* altered in 90 (31%) of 291 patients/cases

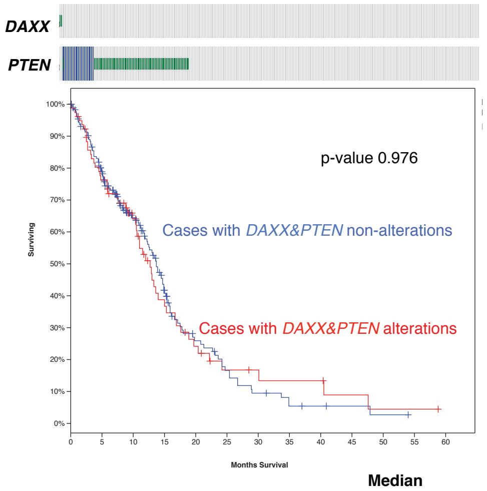

	Total cases	Cases deceased	Median months survival
**Cases with alterations**	79	52	12.7
**Cases without alterations**	176	116	13.7

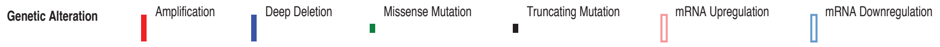

Supplementary Figure 19. DAXX genetic alterations in GBMs. (a) Top panel, OncoPrint image of DAXX alterations in GBMs. Bottom panel, overall survival curve of patients with DAXX mutations compared with non-altered cases. (b) Top panel, OncoPrint image of DAXX and PTEN alterations in GBMs. Bottom panel, overall survival curve of patients with both DAXX and PTEN alterations compared with non-altered cases. Genetic alterations code is indicated at the bottom of the page.

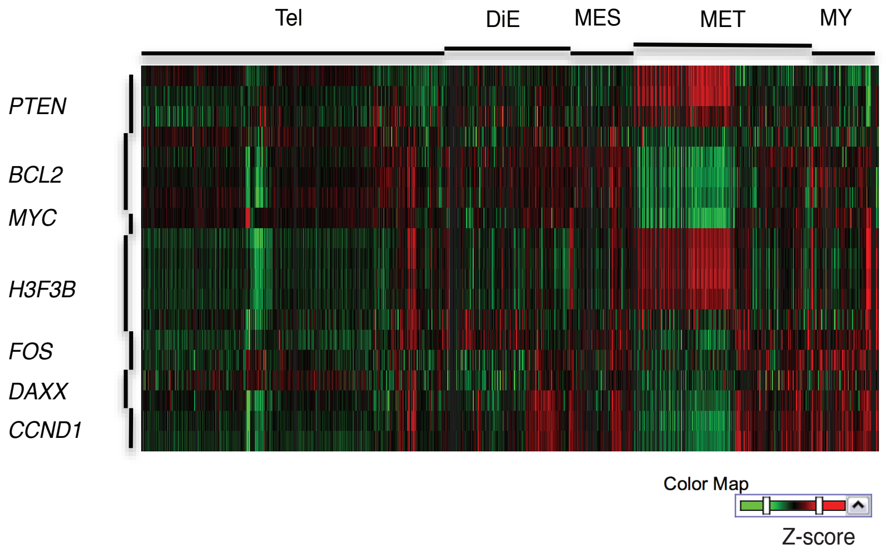

Supplementary Figure 20. PTEN expression is anti-correlated with tumor-driver genes expression in normal human brain. Gene expression analysis of PTEN, H3F3B, BCL2, MYC, FOS and CCND1 using the Allen human brain database. Tel, telencephalon; Die, diencephalon; MES, mesencephalon; MET, metencephalon and MY, myelencephalon.

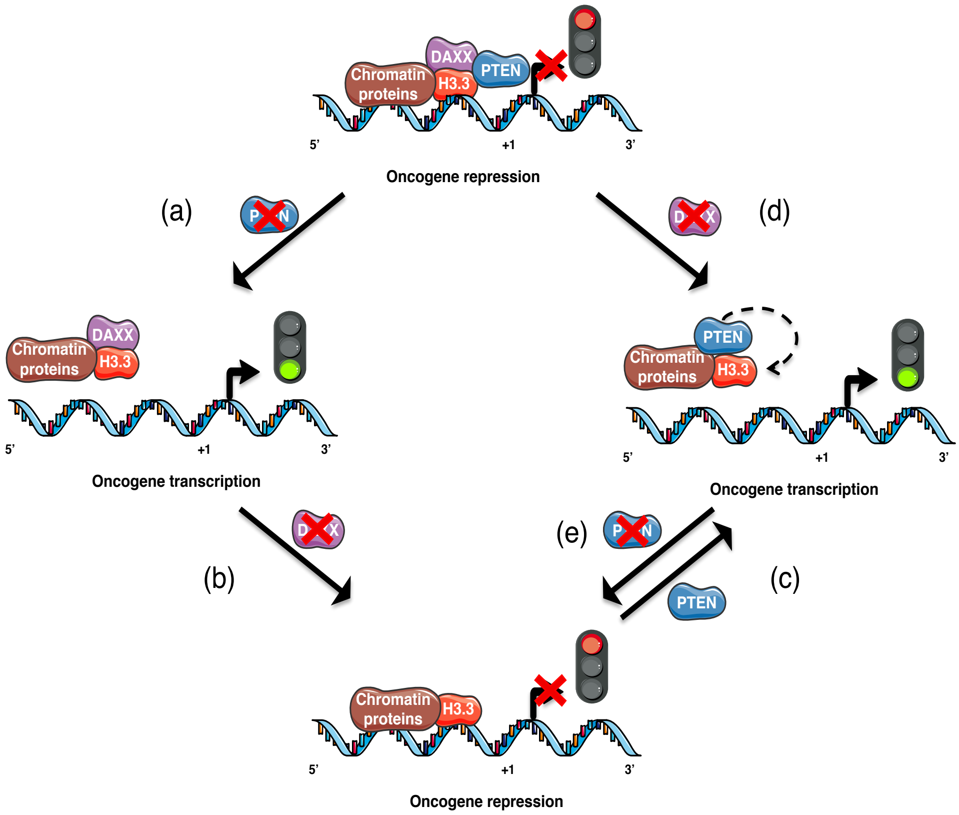

Supplementary Figure 21. PTEN-DAXX-H3.3 complex regulates tumorigenesis. (a) In PTEN deficient tumor cells DAXX removes H3.3 from the chromatin and induces oncogene expression. (b) Inhibition of DAXX restores H3.3 on the chromatin and inhibits oncogene expression. (c) PTEN re-expression in DAXX deficient cells blocks tumor-inhibition effect by interacting with H3.3, (d) as happens in a Daxx-deficient MEF model. (e) PTEN-inhibition in DAXX-null cells represses oncogene expression and reestablishes H3.3 loading on the chromatin.

Figure 1b

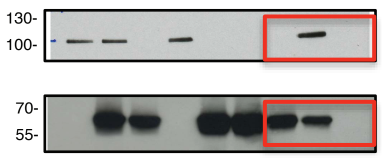

Figure 1d

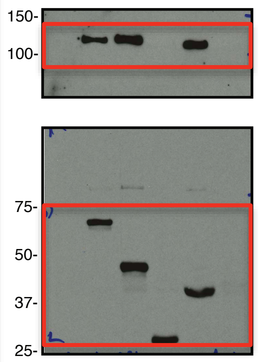

Figure 1e

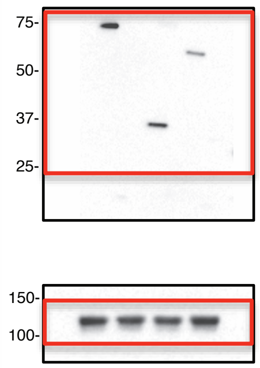

Figure 1c

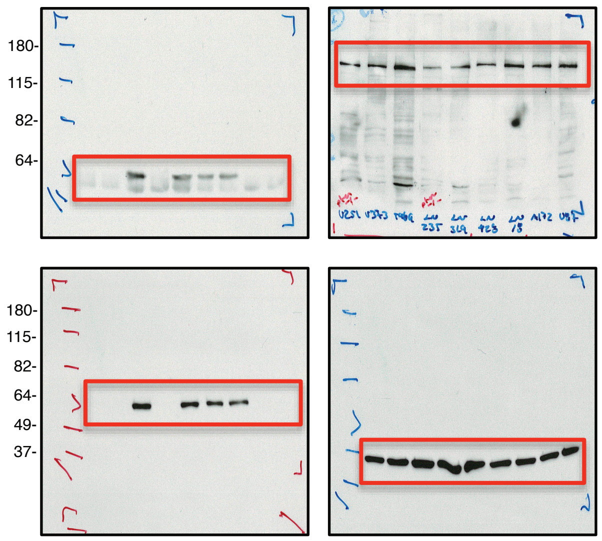

Figure 2a

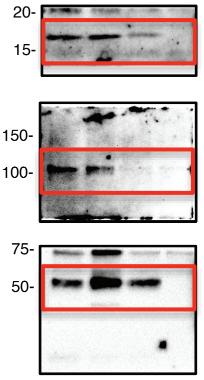

Figure 2b

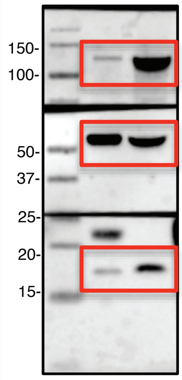

Figure 2d

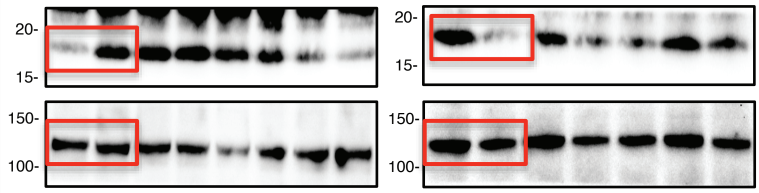

Figure 2i

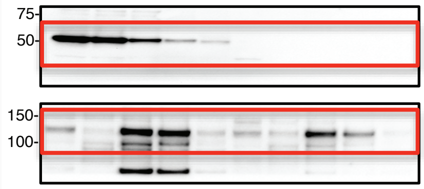

Figure 3a

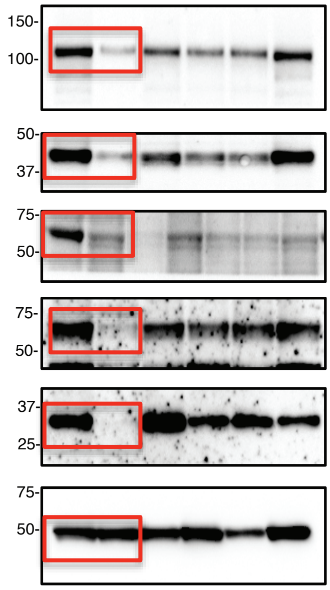

Figure 3b

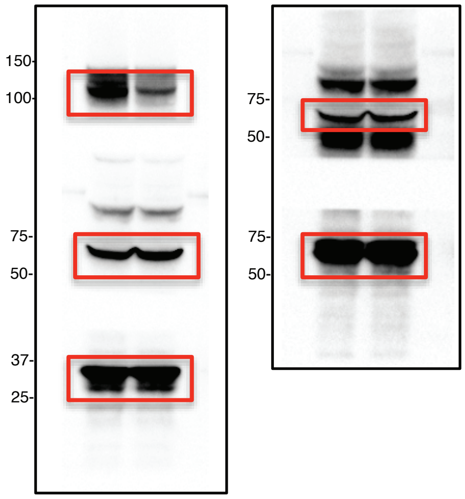

Figure 3f

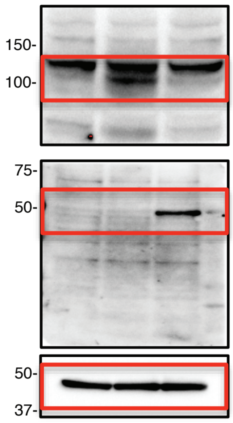

Figure 5j

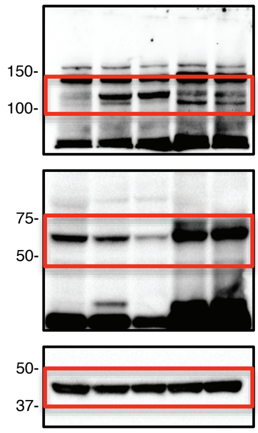

Supplementary Figure 22. Uncropped blots presented in the main paper. Red box indicates the cropped region. Molecular weight is indicated on the side of the blots.

SUPPLEMENTARY METHODS

Antibodies and reagents

Antibodies for western blot: anti-PTEN (7974), and anti-Fos (52), were from Santa Cruz Biotechnology; anti-Lamin A/C (2032), anti-phospho-Akt-Ser (4058), anti-Akt (9272), anti-Myc (5605), anti-CyclinD1 (2926), anti-Bcl2 (2876), and anti-Sox2 (3579) were from Cell Signaling Technology; anti-Histone 3.3 (09-838), anti-Musashi-1 (AB5977) and anti-Olig2 (AB9610) were from Millipore; anti-PAX6 (901301) was from BioLegend; anti-Flag (A8592) and anti-ATRX (HPA001906) were from Sigma-Aldrich; anti-GFP (11-814-460-001) was from Roche; and anti-DAXX (ab32140) was from Abcam.

Antibodies for ChIP assay: anti-Histone 3.3 (17-10245), anti-Trimethyl-Histone H3-Lys27 (17-622), anti-RNA PolII (17-672) were from Millipore.

Antibodies for immunofluorescence: anti-DAXX (8043 and 7152), anti-PTEN-N19 (6818) and anti-ATRX (15408) were from Santa Cruz Biotechnology; anti-Histone 3.3 (09-838) anti-OLIG2 (AB9610) was from Millipore; anti-GFAP (Z0334) was from Dako; anti-HIRA (SAB1400286) was from Sigma; anti-rabbit Alexa Fluor 594 (A21207), anti-mouse Alexa Fluor 488 (A11001), anti-mouse Alexa Fluor 647 (A31571) and anti-goat Alexa Fluor 488 (A21467) were from Life Technologies.

Antibodies for immunocytochemistry: anti-DAXX (HPA008736) was from Sigma-Aldrich; anti-PTEN (9559) from Cell Signaling Technology.

Antibodies for flow cytometry: anti-H2AX-P (9718) from Cell Signaling Technology; anti-RAD52 (8350) and anti-RAD51 (8349) from Santa Cruz Biotechnology; anti-ATRIP-P (ab182626) and anti-RPA32-P (ab2175) from Abcam. Anti-CD133-VioBrightFITC (293C3) was from Macs Miltenyi Biotec.

Kits: Universal magnetic Co-IP kit (54002) and ChIP-IT High Sensitivity (53040) were from Active Motif. Subcellular protein fractionation kit (78840) was from Thermo Scientific.

Reagents: Hygromacin B, Zeocin and Puromycin were from Life Technologies.

Cell culture and plasmids

Daxx +/+ and Daxx -/- moues embryonic fibroblasts (MEFs) were obtained from Philip Leder, Harvard Medical School[1]. Moues embryonic fibroblasts (MEFs) and human glioma cell lines were cultured in DMEM plus 10% of fetal bovine serum (GIBCO/Life Technologies). Human glioblastoma patient-derived (GBM-PDX) spheres were maintained in DMEM/F12 1:1

medium with B27 supplement (GIBCO/Life Technologies) plus human recombinant EGF (20 ng/ml) and FGF (10 ng/ml) (Stemcell Technologies). Genetic alterations and histopathologic classifications of GBM-PDX spheres have been previously described: GBM6 and GBM39 were obtained from David James, Northwestern University[2]; GSC11 and GSC23 were acquired from Frederick Lang, MD Anderson[3]; GBM1600 was kindly provided by Paul Mischel, UCSD[4]; TS528, TS543, TS576 and TS600 were provided by Cameron Brennan, Memorial S Kettering Cancer Center[5]; HK281 and HK157 were obtained from Harley Kornblum, UCLA[6]. All cells were incubated at 37°C, 5% CO_2 and 100% relative humidity. MEF cells and human cell lines were expanded with 0.05% Trypsin (Thermo Scientific); GBM-spheres were dissociated with Accutase (Stemcell Technologies). Cells over-expressing HA-DAXX were generated by electroporation (VPH-1001 Lonza) using 5 μg of pcDNA3-Zeo-HA-Daxx. pcDNA3.1-Daxx vector was kindly provided by Daiqing Liao, University of Florida[7]. DAXX shRNA-resistant construct was generated by nucleotide replacement of the shRNA target sequences that recognize shDaxx4 (*TCACCATCGTTACTGTCAGAA* for *AGCCCGAGCCTGTTAAGCGAG*) and shDaxx5 (*GCCACACAATGCGATCCAGAA* for *GCGACCCAGTGTGACCCGGAG)* clones*.* ATRX-WT was purchased from AddGene (45444) and ATRX-R1426* was generated by PCR cloning (forward primer AGGACGAAACACCGGTCGCAAATGGGCGGTAGGCGTG and reverse primer TCGAGGTCGAGAATTCTTACCTTTTCTTTTTCTGTTTATAGCTCCGCTGA).

In vitro **pull-down assay**

50 ng of human recombinant His-PTEN (ENZO Life Science) proteins were previously immobilized on 30 μl of nickel-beads (GE Healthcare Life Sciences) and then incubated with 200 ng of human recombinant Flag-DAXX protein (OriGene Technologies) for 2 hours at 4°C. Bound proteins were eluted and visualized by immunoblotting.

Immuno-precipitation experiments

For immune-precipitation analysis, whole-cell lysates or nuclear fractions were extracted following the manufacturer's instruction provided with the Universal Magnetic co-IP kit (54002, Active Motif). 300 μg of proteins were immunoprecipitated using 2.0 μg of antibody and 20 μl of Dynabeads (10007D, life technologies).

RNA isolation, reverse transcription and quantitative PCR

Total RNA was isolated with RNeasy kit (Qiagen), quantified and 1 μg of RNA was reverse-transcribed with the Superscript II reagent (Invitrogen). Q-PCR was performed using SYBER Green mix (BioRad). Primers are described below.

Lentivirus production

Mission shRNA lentiviral particles (Sigma-Aldrich) were produced by co-transfection of shRNA pLKO-base lentivirus targeting *Daxx* or shRNA control vector, packaging gene vector pDELTA-8.9 and viral envelope vector pVSV-G in HEK293 cells with Lipofectamine 2000 (Life Technologies). 48 and 72 hours post-transfection supernatant containing lentiviral particles was collected and filtered using 0.45 μm filter (Thermo Scientific). 100 μl of supernatant was used for infection. shRNA sequences are described below. Stable knockdown cells were established by drug selection for 2 weeks after infection.

Lentivirus purification

Supernatants were concentrated by ultracentrifugation at 20,000 rpm for 2 h at 20°C using conical tubes and swinging bucket rotors. Pellets containing viral particles were resuspended in 100 μl of HBSS and 750 μl of 20% sucrose, and centrifuged at 20,000 rpm for 2 h at 20°C. Final pellets were resuspended in 200 μl of HBSS and aliquots of 20 μl were stored at -80°C. Lentiviral titration was determined using p24 ELISA kit according to the manufacturer's protocol (632200, Clontech).

Chromatin immunoprecipitation

Chromatin was isolated from 2 million cells according to manufacturer's recommended procedure in CHIP-IT kit (Active Motif 53040). 250 μg of sheared chromatin was immunoprecipitated using 5 μg of ChIP quality antibody. ChIP-DNA was eluted (200 μl of elution buffer for ChIP-PCR or in 50 μl for ChIP-seq), and 2 μl were analyzed by q-PCR using SYBER Green mix (BioRad). 25 μg of sheared chromatin were used as Input-DNA. Primers were designed by Prime3 and validated in the Genome Browser. Primers are described below.

Cellular fractionation

Subcellular protein fractions were extracted according to manufacturer's instruction (Thermo Scientific) and 10 μg of proteins were resolved by SDS-PAGE followed by immunoblotting.

Immunofluorescence microscopy

Cells were plated on poly-d-lysine-coated glass coverslips (Thermo Scientific), fixed with 10% formalin (Sigma-Aldrih), blocked with 2% of BSA IgG-free (Jackson ImmunoResearch) and stained with primary antibodies overnight at 4°C. Secondary antibody was added for 1h at room temperature. Coverslips were mounted on microscope glass slides using Fluro-Gel with DAPI (Electron Microscopy Science) followed by visualization using confocal microscopy (Leica SP5 confocal with resonant scanner).

Immunofluorescence of proteins associated to chromatin in GBM-PDX neurospheres was performed according to Forment and Jackson, 2015[8]. Briefly, cells were resuspended in extraction buffer (10 mM PIPES pH 7.0, 100 mM NaCl, 3 mM MgCl2, 300 mM sucrose and 0.7% Triton X-100; plus 0.3 g/ml RNase A), incubated on ice for 10 minutes, washed with 1% PBS-BSA and centrifuged 10 minutes at 800g at 4˚C. Then, cells were fixed with fixation buffer (2% paraformaldehyde in PBS) at room temperature for 30 minutes, washed and centrifuged at 4˚C. Cells were incubated with primary antibodies 1:50 overnight at 4˚C, washed and centrifuged at 4˚C. Finally, incubated with secondary antibodies 1:1000 at room temperature for 30 minutes, resuspended with mounting solution and pipetted onto glass slides (Fluro-Gel with DAPI Electron Microscopy Science). Colocalization analysis and statistics values (Person's coefficient) were obtained using Imaris8, Bitplane software.

Cell proliferation and cell cycle analysis

1,000 cells were grown in 96-well plates and 72 hours later cell proliferation was analyzed by WST1 (MK400, Clontech) or ATplite assay (6016941, Perkin-Elmer). For cell cycle progression, cells were fixed with 70% of ethanol overnight and stained with FxCycle PI/RNase staining solution (F10797, Life Technologies). DNA content was evaluated by flow cytometry (FACS).

Promoter reporter assay

A 1.5 and 1.0 Kb fragment upstream of the transcriptional start site of the human *CCND1* and *MYC* genes, respectively, were cloned into the pLightSwicth vector (32001, Active Motif). shControl, shDaxx knockdown and PTEN-WT overexpressing cells were transfected with pLightSwicth vector or GAPDH-pLightSwicth vector (32004, Active Motif) using FuGene-HD (F200, Active Motif). 48h post-transfection luciferase reporter signal was determined using LightSwitch Luciferase Assay Reagent LS100 (32032, Active Motif). Four replicates per transfection per condition were analyzed.

Sphere formation assay

Glioma stem cells were dissociated into single cells and 100 or 500 cells/well were plated in 96-well plates. Total number of spheres and total number of cells, per well and per treatment, were determined after 14 days in culture.

***In vitro* limiting dilution assay**

Glioma spheres were dissociated into single cells and 1, 5, 10, 20, 50 and 100 cells/well were plated in 96-well plates with five replicates for each experimental condition. The total number of spheres, per well and per treatment were quantified after 14 days in culture. Data was analyzed by extreme limiting dilution analysis (ELDA, http://bioinf.wehi.edu.au/software/elda/)[9].

Flow cytometry analysis

Quantification of proteins associated to the fork replication complex was determined by flow cytometry according to Forment and Jackson, 2015[8]. As positive control cells were treated with 2mM of Hydorxyurea (HU) for 24 hours and markers of the replication for complex were analyzed H2AX-P, RAD52, RAD51, ATRIP-P and RPA32-P[10-12].

Differentiation of GBM-PDX spheres

GBM spheres were dissociated into single cells and plated on glass coverslips coated with poly-d-lysine in DMEM medium with 1% of FBS. Coverslips were processed for immunostaining 7 days after plating.

Lentivirus transduction of GBM-PDX spheres

Glioma spheres were co-transduced with purified lentivirus that encoded shRNAs anti-DAXX (shDAXX) or shRNA control (shLuc) at multiplicity of infection (MOI) 5, and a near infrared fluorescence protein (IRFP720, PerkinElmer) MOI 5, for 96 hours.

Intracranial xenograft tumor model

Animal research experiments were conducted under the regulations of the UCSD Animal Care Program, protocol number S00192M. GBM-PDX spheres were harvested and resuspended at 0.5 or $1.0x10^6$ cells in $2\mu l$ of PBS per animal, then stereotactically injected into the striatum (1.0 mm anteroposterior and 2.0 lateral from Bregma suture and 3mm below the pial surface) of immunodeficient mice (Charles River laboratory).

Tumor size measurement and survival analysis

Animals were observed for neurological signs and the relative fluorescence signal of the xenografts were analyzed by fluorescence molecular tomography (FMT, PerkinElmer) and quantified using TrueQuant 3.1 software (PerkinElmer). For survival analysis, animals were euthanized when they showed signs of distress and morbidity.

Densitometry quantification

Immunoblots were acquired with ChemiDocMP (BioRad) and the intensity signal was quantified by densitometry analysis with Image Lab software.

Immunohistochemistry

Slides were deparaffinized and rehydrated by washing steps of 3 minutes in xylene, xylene:ethanol 1:1, 100% ethanol, 95% ethanol, 70% ethanol, 50% ethanol and water. After deparaffinization, sections were boiled in citrate buffer (pH 6.0) for 25 minutes. Sections were then treated with 5% serum-blocking solution for 20 minutes. Primary rabbit-anti-DAXX antibody (HPA008736, Sigma) was diluted 1:200 and incubated overnight at 4°C. Sections were washed twice with TBS-tween before incubation with 3% hydrogen peroxide for five minutes. After washing three times, sections were incubated for 1 h with a biotinylated goat-anti-rabbit secondary antibody (Vector Laboratories). After washing two times, sections were incubated with ABC-complex (Vector Laboratories) for 30 minutes and

finally treated with DAB (3,3'-Diaminobenzidine) for 1 minute. Sections were analyzed on a Nikon light microscope using Nikon imaging software.

Tissue microarray

Paraffin sections from a Tissue microarray of 100 GBM patients were prepared for immunostaining with anti-DAXX and anti-PTEN primary antibodies. Only 6 out of 100 samples were IDHmt (R132M) positive based on IHC analysis. 67 cases had sufficient material left for analyses of all markers. Scoring of stained sections was performed independently by two observers (MA and HM). Scoring scheme: Intensity of staining: negative (0); weak (1); moderate (2); strong (3). The mean scoring was assessed from both observers and served as the final score displayed in the results section. The Norwegian Data Inspectorate and the Regional Committee for Ethics in Research have approved this project. The study was performed in accordance with the Helsinki Declaration.

In silico protein-protein interactions

New PTEN nuclear interacting complexes were simulated using the bioinformatics site Human Interactome Map [13]. Briefly, HiMAP bioinformatic source was seeded with protein of interest PTEN and with previously described proteins that interact with PTEN such as TP53[14], PML[15] and MCRS1[16]. HiMAP analysis revealed that PTEN was predicted to interact with DAXX, which has been reported to interact with PML[17], TP53[7] and MCRS1[18].

Chromatin Immunoprecipitation sequencing (ChIP-seq) and analysis

Libraries were made with the Kapa Hyper Prep kit (Roche, KK8502), starting with 2.5 ng of IP DNA, ad amplified by 15 cycles pf PCR amplification, according to the manufacturer's protocol. Libraries were quantified and sized by running them on an Agilent Tapestation, measuring concentration of QPCR (Kapa Universal Library Quantification kit, Roche, KK4824). The libraries were run on an Illumina 2500, v4 chemistry, using a single read 50 protocol.

ChIP-Seq analysis was performed using the HiC-Bench ("HiC-Bench," https://github.com/NYU-BFX/hic-bench) pipeline software package developed by the Applied Bioinformatics Center. Sequencing read and alignment quality metrics were

determined using R (R Core Team 2016 https://www.R-project.org/), DeepTools[19], SAMtools[20], BEDtools[21], Picard Tools ("Picard Tools" 2013, http://broadinstitute.github.io/picard/), BigWig and BigBed tools[22], and GenomicTools[23]. Raw reads were aligned to the hg19 reference genome using bowtie2[24]. Peaks were called with MACS2[25], and examined with the UCSC Genome Browser[26]. Differential binding patterns were analyzed with DiffBind[27]and R (R Core Team 2016). Gene ontology profiles and KEGG pathways were determined with the clusterProfiler[28] software package. This work used computing resources at the Laura and Isaac Perlmutter Cancer Center, which is supported by Cancer Center Support Grant P30CA016087. A Tsirigos was supported by a Research Scholar Grant, RSG-15-189-01-RMC from the American Cancer Society. This work also used computing resources at the High Performance Computing Facility of the Center for Health Informatics and Bioinformatics at the NYU Langone Medical Center.

RNA sequencing (RNA-seq) and analysis

Total RNA was assessed for quality using an Agilent Tapestation, and all samples had RNA Integrity Numbers (RIN) above 9.0. RNA libraries were generated using Illumina's TruSeq Stranded mRNA Sample Prep Kit (Illumina, RS-122-2101) following manufacturer's instructions, modifying the shear time to 5 minutes.

We aligned RNA-seq reads to the human genome (hg19) with STAR 2.4.0h (outFilterMultimapNmax 20, outFilterMismatchNmax 999, outFilterMismatchNoverLmax 0.04, outFilterIntronMotifs RemoveNoncanonicalUnannotated, outSJfilterOverhangMin 6 6 6 6, seedSearchStartLmax 20, alignSJDBoverhangMin 1) using a gene database constructed from Gencode v19 [29,30]. We counted reads that overlap with exon coordinates using HTSeq-count (-s reverse -a 0 -t exon -i gene_id -m union) [31,32]. Raw read counts were processed with DESeq2 [33] and only genes with mean read count > 20 were considered for the analysis.

Raw read counts were transformed using the variance stabilizing transformation (VST) function included in DESeq2 [34]. Mean and standard deviation of normalized expression were calculated for each gene and Z-scores were determined by subtracting the mean from each expression value and dividing by the standard deviation. Z-scores were used to build Figure

4e and Supplementary Figure 9. Oncogenes and tumor suppressors were derived from the Cancer Gene Census[35].

TCGA and REMBRANDT analysis

Gene expression, genetic alterations and survival rate in GBMs from TCGA data were analyzed using the cBioPortal, http://www.cbioportal.org/index.do[36,37]. Gene expression comparison in all gliomas was determined using REMBRANDT database, http://www.betastasis.com. Spatial and gene expression analysis in human brain were performed using the Allen Human Brain Atlas, http://human.brain-map.org/ [38].

The normalized expression levels of 20,502 human genes in the 166 GBM samples derived from RNA-seq data were downloaded from the TCGA (https://tcga-data.nci.nih.gov/tcga/). Correlation between the expression levels of *PTEN* and *DAXX* was calculated for the 48 samples with mutated *PTEN* and for the 118 samples with wild-type *PTEN* separately. Correlation tests were calculated using the cor.test function in R. Correlation between *DAXX* and each of the other 20,501 human genes was calculated considering all 166 GBM samples. These 20,501 human genes were ranked on the basis of the correlation with *DAXX* expression, from most positively correlated to the most anti-correlated (Supplementary table 1). The ranked list was used as input for Gene Set Enrichment Analysis (GSEA)[39,40]. 710 gene sets, including 50 hallmark[41], 148 Biocarta (http://www.biocarta.com/), and 512 Reactome[42] gene sets, were downloaded from the Molecular Signature Database (MSigDB)[40] and used to run GSEA with default parameters (1,000 permutations, gene set size between 15 and 500 genes).

Correlation between expression levels of *PTEN* and *DAXX* was calculated also for 1,096 breast cancers. This analysis was performed separately for tumor with wild-type *PTEN* (1,066 breast cancers) and tumors with mutated *PTEN* (30 breast cancers).

Data availability

Data generated during the study have been deposited in Sequence Read Archive (SRA) SRP090820.

Statistical analysis

Data sets were analyzed by unpaired t-test or multiple comparisons one-way ANOVA or two-way ANOVA according to the experiment using GraphPad Prism software. *p<0.05, **p<0.001 and ***p<0.0001. Kaplan-Meier curves and comparison of survival were analyzed using Long-rank (Mantel-Cox) test.

Supplementary Table 1

Sequence Name	Sequence
CHIP-hCCND1-P1-F	CTC CAC CTC ACC CCC TAA AT
CHIP-hCCND1-P1-R	GGG GGC GGG CGC AGG GGG A
CHIP-hCCND1-P2-F	TCC CAT TCT CTG CCG GGC TTT GAT C
CHIP-hCCND1-P2-R	GCT GGT GTT CCA TGG CTG GGG C
CHIP-hCCND1-P3-F	CAT AAA TCA TCC AGG CGG CC
CHIP-hCCND1-P3-R	ATA GCC AAG CCT CAG AGC AT
CHIP-hCCND1-P4-F	TCA GAG GTG TGT TTC TCC CG
CHIP-hCCND1-P4-R	GCC TTC CTA CCT TGA CCA GT
CHIP-hMYC-P1-F	CCC AAA AAA AGG CAC GGA A
CHIP-hMYC-P1-R	TAT TGG AAA TGC GGT CAT GC
CHIP-hMYC-P2-F	ACC AAA TAC CCA TCA CCT TCT G
CHIP-hMYC-P2-R	CAA ACC CTA AAA CGG CCA AAC
CHIP-hMYC-P3-F	GCT GCA AAC TCA ACG GGT AA
CHIP-hMYC-P3-R	CCT CCA CCA CCT CCA AAA GA
CHIP-hMYC-P4-F	GGG TAC AGA CTG GCA GAG AG
CHIP-hMYC-P4-R	GCG TCT GTT TAG CCC TGA GA
CHIP-hBcl2-P1-F	GGC TCA GAG GAG GGC TCT TT
CHIP-hBcl2-P1-R	GTG CCT GTC CTC TTA CTT CAT TCT C
CHIP-hBcl2-P2-F	GTG TTC CGC GTG ATT GAA GAC
CHIP-hBcl2-P2-R	CAG AGA AAG AAG AGG AGT TAT AA
CHIP-hBcl2-P3-F	CCA GGC AGC TTA ATA CAT TCT TTT TAG
CHIP-hBcl2-P3-R	TGA TGC TGA AAG GTT AAA GAA AAA AC
CHIP-hFOS-P1-F	CAG ATT TGA GTT CCC CGC AG
CHIP-hFOS-P1-R	GTG CGA GGT GTG GTT ATG TG
CHIP-hFOS-P2-F	CAC ATA ACC ACA CCT CGC AC
CHIP-hFOS-P2-R	GCA AAA CCT ACG TGC GAA GA
CHIP-hFOS-P3-f	GTT GAG CCC GTG ACG TTT AC
CHIP-hFOS-P3-R	AGA TGC GGT TGG AGT ACG AG
CHIP-mCcnd1-P1-F	ACC CCG AAA ATT CCA GCA AC

CHIP- mCcnd1-P1-R	AAT CTC AGA TCC CAC CCC AC
CHIP- mCcnd1-P12-F	TAC CCG ACT TCA AGC TAG GC
CHIP- mCcnd1-P2-R	GTG CGC TCC TTT ACC AGT TT
CHIP- mCcnd1-P3-F	CTT CTG CAC GCA CTT GAA GT
CHIP- mCcnd1-P3-R	CCC TGA CAC CAA TCT CCT CA
CHIP-mMyc-P1-F	CAG AGT CTG CTG CAA ACT GG
CHIP-mMyc-P1-R	CCC ATA CAC CTC CAC ACA GT
CHIP-mMyc-P2-F	TGC GGT GAC TGA TAT ACG CA
CHIP-mMyc-P2-R	GCG CTA GTC CTT TCC CTT TC
CHIP-mMyc-P3-F	AAA GAA GGG AGG GGA GGG AT
CHIP-mMyc-P3-R	GCT CAC TCC CTC TGT CTC TC
CHIP-mBcl2-P1-F	AGC AGC CAG AGA GAT TAC CG
CHIP-mBcl2-P1-R	AGT TGC CTC TCT TCA CCC AA
CHIP-mBcl2-P2-F	TTA CAC TTG CAC ACA CAC GC
CHIP-mBcl2-P2-R	GTG TCT CTC TGC CCT GGA G
CHIP-mBcl2-P3-F	TTC CTC TGC TCC TAA CGC TC
CHIP-mBcl2-P3-R	CCA AAA CCG TAC CTA TGC CG

Human and mouse primers for ChIP-PCR.

Supplementary Table 2

Sequence Name	Sequence
RT-hCCND1-F	CCA TTC CCT TGA CTG CCC GAG
RT-hCCND1-R	GAC CAG CCT CTT CCT CCA C
RT-hMYC-F	TTC GGG TAG TGG AAA ACC AG
RT-hMYC-R	AGT AGA AAT ACG GCT GCA CC
RT-hBCL2-F	GTG GAT GAC TGA GTA CCT GAA C
RT-hBCL2-R	GCC AGG AGA AAT CAA ACA GAG G
RT-hGAPDH-F	GGA AGG TGA AGG TCG GAG TCA
RT-hGAPDH-R	GTC ATT GAT GGG AAC AAT ATC
RT-mFos-F	CCAGTCAAGAGCATCAGCAA
RT-mFos-R	AAGTAGTGCAGCCCGGAGTA
RT-mMyc-F	GCCCAGTGAGGATATCTGGA
RT-mMyc-R	ATCGCAGATGAAGCTCTGGT
RT-mBcl2-F	CTGGCATCTTCTCCTTCCAG
RT-mBcl2-R	GACGGTAGCGACGAGAGAAG
RT-mCcnd1-F	AGTGCGTGCAGAAGGAGATT
RT-mCcnd1-R	CACAACTTCTCGGCAGTCAA

Human and mouse primers for RT-PCR.

Supplementary Table 3

shRNA	Clone ID	Sequence
human shDaxx3	TRCN0000003800	CGGGAAGGGATGGACTAAGCTAATCTCGAGATTAGCTTAGTCCATCCCTTCTTTTT
human shDaxx4	TRCN0000003801	CCGGTCACCATCGTTACTGTCAGAACTCGAGTTCTGACAGTAACGATGGTGATTTTT
human shDaxx5	TRCN0000003802	CCGGGCCACACAATGCGATCCAGAACTCGAGTTCTGGATCGCATTGTGTGGCTTTTT
mouse shDaxx3	TRCN0000077385	CCGGGCGCATTGAACGGCTCATTAACTCGAGTTAATGAGCCGTTCAATGCGCTTTTTG
mouse shDaxx4	TRCN0000077386	CCGGCCTGGATCTCATCTACAACTTCTCGAGAAGTTGTAGATGAGATCCAGGTTTTTG
mouse shDaxx5	TRCN0000077387	CCGGCCGGAGGAGATCATCGTGCTTCTCGAGAAGCACGATGATCTCCTCCGGTTTTTG

Human and mouse shRNA sequences for DAXX knockdown.

SUPPLEMENTARY REFERENCES

1. Michaelson, J.S., Bader, D., Kuo, F., Kozak, C. & Leder, P. Loss of Daxx, a promiscuously interacting protein, results in extensive apoptosis in early mouse development. *Genes Dev* **13**, 1918-1923 (1999).

2. Sarkaria, J.N.*, et al.* Use of an orthotopic xenograft model for assessing the effect of epidermal growth factor receptor amplification on glioblastoma radiation response. *Clin Cancer Res* **12**, 2264-2271 (2006).

3. Jiang, H.*, et al.* Examination of the therapeutic potential of Delta-24-RGD in brain tumor stem cells: role of autophagic cell death. *J Natl Cancer Inst* **99**, 1410-1414 (2007).

4. Nakano, I.*, et al.* Siomycin A targets brain tumor stem cells partially through a MELK-mediated pathway. *Neuro Oncol* **13**, 622-634 (2011).

5. Inda, M.M.*, et al.* Tumor heterogeneity is an active process maintained by a mutant EGFR-induced cytokine circuit in glioblastoma. *Genes Dev* **24**, 1731-1745 (2010).

6. Visnyei, K.*, et al.* A molecular screening approach to identify and characterize inhibitors of glioblastoma stem cells. *Mol Cancer Ther* **10**, 1818-1828 (2011).

7. Zhao, L.Y.*, et al.* Negative regulation of p53 functions by Daxx and the involvement of MDM2. *J Biol Chem* **279**, 50566-50579 (2004).

8. Forment, J.V. & Jackson, S.P. A flow cytometry-based method to simplify the analysis and quantification of protein association to chromatin in mammalian cells. *Nat Protoc* **10**, 1297-1307 (2015).

9. Hu, Y. & Smyth, G.K. ELDA: extreme limiting dilution analysis for comparing depleted and enriched populations in stem cell and other assays. *J Immunol Methods* **347**, 70-78 (2009).

10. Zou, L. & Elledge, S.J. Sensing DNA damage through ATRIP recognition of RPA-ssDNA complexes. *Science* **300**, 1542-1548 (2003).

11. Murphy, A.K.*, et al.* Phosphorylated RPA recruits PALB2 to stalled DNA replication forks to facilitate fork recovery. *J Cell Biol* **206**, 493-507 (2014).

12. Petermann, E., Orta, M.L., Issaeva, N., Schultz, N. & Helleday, T. Hydroxyurea-stalled replication forks become progressively inactivated and require two different RAD51-mediated pathways for restart and repair. *Mol Cell* **37**, 492-502 (2010).

13. Rhodes, D.R.*, et al.* Probabilistic model of the human protein-protein interaction network. *Nat Biotechnol* **23**, 951-959 (2005).

14. Freeman, D.J., *et al.* PTEN tumor suppressor regulates p53 protein levels and activity through phosphatase-dependent and -independent mechanisms. *Cancer Cell* **3**, 117-130 (2003).

15. Song, M.S., *et al.* The deubiquitinylation and localization of PTEN are regulated by a HAUSP-PML network. *Nature* **455**, 813-817 (2008).

16. Okumura, K., Zhao, M., Depinho, R.A., Furnari, F.B. & Cavenee, W.K. Cellular transformation by the MSP58 oncogene is inhibited by its physical interaction with the PTEN tumor suppressor. *Proc Natl Acad Sci U S A* **102**, 2703-2706 (2005).

17. Zhong, S., *et al.* Promyelocytic leukemia protein (PML) and Daxx participate in a novel nuclear pathway for apoptosis. *J Exp Med* **191**, 631-640 (2000).

18. Lin, D.Y. & Shih, H.M. Essential role of the 58-kDa microspherule protein in the modulation of Daxx-dependent transcriptional repression as revealed by nucleolar sequestration. *J Biol Chem* **277**, 25446-25456 (2002).

19. Ramirez, F., *et al.* deepTools2: a next generation web server for deep-sequencing data analysis. *Nucleic Acids Res* **44**, W160-165 (2016).

20. Li, H., *et al.* The Sequence Alignment/Map format and SAMtools. *Bioinformatics* **25**, 2078-2079 (2009).

21. Quinlan, A.R. & Hall, I.M. BEDTools: a flexible suite of utilities for comparing genomic features. *Bioinformatics* **26**, 841-842 (2010).

22. Kent, W.J., Zweig, A.S., Barber, G., Hinrichs, A.S. & Karolchik, D. BigWig and BigBed: enabling browsing of large distributed datasets. *Bioinformatics* **26**, 2204-2207 (2010).

23. Tsirigos, A., Haiminen, N., Bilal, E. & Utro, F. GenomicTools: a computational platform for developing high-throughput analytics in genomics. *Bioinformatics* **28**, 282-283 (2012).

24. Langmead, B. Aligning short sequencing reads with Bowtie. *Curr Protoc Bioinformatics* **Chapter 11**, Unit 11 17 (2010).

25. Feng, J., Liu, T., Qin, B., Zhang, Y. & Liu, X.S. Identifying ChIP-seq enrichment using MACS. *Nat Protoc* **7**, 1728-1740 (2012).

26. Kent, W.J., *et al.* The human genome browser at UCSC. *Genome Res* **12**, 996-1006 (2002).

27. Ross-Innes, C.S., *et al.* Differential oestrogen receptor binding is associated with clinical outcome in breast cancer. *Nature* **481**, 389-393 (2012).

28. Yu, G., Wang, L.G., Han, Y. & He, Q.Y. clusterProfiler: an R package for comparing biological themes among gene clusters. *OMICS* **16**, 284-287 (2012).

29. Dobin, A., *et al.* STAR: ultrafast universal RNA-seq aligner. *Bioinformatics* **29**, 15-21 (2013).

30. Harrow, J., *et al.* GENCODE: the reference human genome annotation for The ENCODE Project. *Genome Res* **22**, 1760-1774 (2012).

31. Anders, S., Pyl, P.T. & Huber, W. HTSeq-a Python framework to work with high-throughput sequencing data. *Bioinformatics* **31**, 166-169 (2015).

32. Anders, S., Reyes, A. & Huber, W. Detecting differential usage of exons from RNA-seq data. *Genome Res* **22**, 2008-2017 (2012).

33. Love, M.I., Huber, W. & Anders, S. Moderated estimation of fold change and dispersion for RNA-seq data with DESeq2. *Genome Biol* **15**, 550 (2014).

34. Anders, S. & Huber, W. Differential expression analysis for sequence count data. *Genome Biol* **11**, R106 (2010).

35. Futreal, P.A., *et al.* A census of human cancer genes. *Nat Rev Cancer* **4**, 177-183 (2004).

36. Gao, J., *et al.* Integrative analysis of complex cancer genomics and clinical profiles using the cBioPortal. *Sci Signal* **6**, pl1 (2013).

37. Cerami, E., *et al.* The cBio cancer genomics portal: an open platform for exploring multidimensional cancer genomics data. *Cancer Discov* **2**, 401-404 (2012).

38. Hawrylycz, M.J., *et al.* An anatomically comprehensive atlas of the adult human brain transcriptome. *Nature* **489**, 391-399 (2012).

39. Mootha, V.K., *et al.* PGC-1alpha-responsive genes involved in oxidative phosphorylation are coordinately downregulated in human diabetes. *Nat Genet* **34**, 267-273 (2003).

40. Subramanian, A., *et al.* Gene set enrichment analysis: a knowledge-based approach for interpreting genome-wide expression profiles. *Proc Natl Acad Sci U S A* **102**, 15545-15550 (2005).

41. Liberzon, A., *et al.* The Molecular Signatures Database (MSigDB) hallmark gene set collection. *Cell Syst* **1**, 417-425 (2015).

42. Croft, D., *et al.* The Reactome pathway knowledgebase. *Nucleic Acids Res* **42**, D472-477 (2014).
